# Climatic Drivers of Flowering Synchrony in ‘Hass’ Avocado Under Tropical Andean Conditions

**DOI:** 10.3390/plants14243822

**Published:** 2025-12-16

**Authors:** Alexander Rebolledo, Ronal Burbano, Jairo Villamizar, Diego Corrales

**Affiliations:** 1 Centro de Investigación Palmira, Corporación Colombiana de Investigación Agropecuaria-Agrosavia, Diagonal a la Intersección de la Carrera 36A con Calle 23, Palmira 763531, Valle del Cauca, Colombia; rburbano@agrosavia.co (R.B.); javillamizar@agrosavia.co (J.V.); 2 Research Unit ITAV: Innovaciones Tecnológicas para Agregar Valor a Recursos Agrícolas, Centro de Investigación La Selva, Corporación Colombiana de Investigación Agropecuaria-Agrosavia, Km. 7, vía Rionegro-Las Palmas, Sector Llanogrande, Rionegro 054048, Antioquia, Colombia; 3 Departamento de Investigación, C.I GLOBAL GREEN S.A.S., Pijao 632060, Quindío, Colombia; dcorrales@dvssas.com

**Keywords:** altitudinal phenology, thermal–hydric gradient, ‘hass’ avocado, phenological synchrony, growing degree days (GDD)

## Abstract

Understanding how climatic variability shapes the reproductive behavior of perennial crops is essential for improving their adaptation to tropical mountain environments. This study examined the influence of altitudinal and thermal gradients on flowering synchrony in ‘Hass’ avocado (*Persea americana* Mill.) cultivated across the tropical Andes of Colombia. Climatic variables and phenological stages were monitored across three elevations (2056, 2212, and 2338 m.a.s.l.) during two production cycles. Principal component, confirmatory factor, and circular statistical analyses were applied to integrate multivariate climatic structure with temporal flowering patterns. Results revealed that temperature was the main climatic driver of phenological variability, with significant differences among altitudes. The study revealed an altitudinal thermal–hydric gradient structuring distinct microclimates between 2050 and 2350 m.a.s.l., which determine the synchrony and rate of ‘Hass’ avocado phenological processes. Anthesis was the most environmentally sensitive phase, showing greater stability at intermediate elevations (~2200 m). Multivariate (PCA, CFA, ANOVA) and circular analyses confirmed that accumulated temperature (GDD) effectively predicts phenological progression, defining an optimal altitudinal range for synchrony and productivity in high Andean temperate zones.

## 1. Introduction

The ‘Hass’ avocado cultivar continues to grow stronger worldwide, reaching figures that exceed 80% of the global trade in the fruit [1]. In Colombia, nearly 98,000 tons were exported in 2022, with an increase of 26.3% for 2023, exceeding the threshold of 120,000 tons [2]. The ‘Hass’ avocado continues to stand out for its high nutritional quality, good productivity, post-harvest life, and wide acceptance in different markets, with the possibility of being produced under various environmental conditions [3,4,5]. Although most avocado-producing areas are located in subtropical climates, the expansion of cultivation to high-altitude tropical areas such as Colombia, which has altitudes ranging from 1600 to 2500 m.a.s.l (meters above sea level) [6,7,8], has generated new agronomic challenges due to the contrasting environmental conditions that prevail there and the need to understand the crop’s responses in this wide range of microclimates [8,9].

The phenology of the ‘Hass’ cultivar is characterized by its complexity due to an exceptionally long reproductive cycle and the marked overlap between the stages of fruit development and flowering of the next harvest. Studies conducted in Mexico showed fruit ripening times of 8 and 12 months with intense competition for energy resources due to the overlap between fruit growth and the induction of new reproductive buds [10].This physiological competition is the underlying cause of alternate bearing, a phenomenon that manifests itself as a biennial cycle of abundant harvests (‘on years’) followed by poor harvests (‘off years’), compromising yield stability. This phenomenon is attributed to competition for photoassimilates and nutritional reserves between the developing fruit and floral differentiation for the next harvest [11].

In this context, phenological synchrony is a critical factor in flower anthesis, since high synchrony enhances fruit set efficiency, a process that is already inefficient due to the synchronized dichogamy of the flower and the low fruit set rate [12]. Characterizing phenological synchrony is essential for understanding the dynamics of yield by altitudinal range or microclimate, since identifying behavior from floral induction to anthesis makes it possible to determine the temporal coherence of the reproductive phases, which is crucial for maximizing pollination efficiency and subsequent fruit set [13].

The flowering periods in subtropical areas are related to the spring season after a stress condition of low temperature. The pattern of floral gene expression associated with low temperature was reported for ‘Hass’ avocado, finding results that suggest ‘Hass’ avocado floral development is promoted by low temperature-dependent floral induction and warm temperature-dependent floral organogenesis [14]. Temperature is known to be a factor that determines the transition from closed pointed bud to reproductive growth and values below 20 °C were enough to induce flowering for the varieties Choquette, Booth-8 and ‘Hass’ in Mexico [10,15].

In tropical environments, temperature variations throughout the year are small and the photoperiod is almost constant, which can modify the environmental signals that regulate floral induction and reproductive development compared to what is observed in subtropical regions, resulting in alterations in flowering cycles and harvest times [9,16]. Other studies mention that in equatorial regions, the length of the day is approximately 12 h throughout the year, so the photoperiod varies little and is not always the main trigger for flowering in tropical species. Therefore, the synchrony of flowering may depend on subtle signals such as variations in (sunrise/sunset), minimum temperature, and precipitation [17].

Avocado productive areas in Colombia have a small annual temperature range that is characteristic of tropical climates, with conditions rarely exceeding 4 °C between the warmest and coldest months. The annual temperature range becomes larger on latitudes further from the Equator, but even at the limits of the tropical zone, few locations have an annual range of more than 15 °C [16,18]. Given the minimal temperature variation and irregular rainfall patterns characteristic of tropical climates, understanding the environmental factors that trigger flowering is crucial for optimizing orchard management. Recent studies of the ‘Hass’ cultivar under variable altitude conditions have shown that phenological stages overlap throughout the year due to temperature and precipitation conditions [9]. Other studies conducted in Colombia suggest that water deficits (low precipitation and high evapotranspiration) during flowering can act as a stress factor for flower abscission [19].

In the ‘Hass’ cultivar, the constant dependence of phenology on temperature takes on critical importance in the context of global climate change and the increase in extreme temperature variability events, which are affecting phenology, pollination, and yield in avocado crops [20]. Global warming trends not only alter the ideal temperature averages for flowering and fruit set, but in mountain ecosystems such as the Andes, they intensify microclimatic gradients, forcing changes in the areas suitable for avocado production [21]. Recent research in Egypt proposes adaptation measures for Fuerte cultivars, focusing efforts on developing irrigation management practices, biochar supply, soil amendments, and the selection of new areas to reduce climate risk [22].

The phenology of the ‘Hass’ cultivar has been studied using various modeling approaches that seek to explain how environmental factors and physiological processes interact to determine the onset and progression of the stages of development from latent bud, anthesis, fruit set, and fruit development. These models range from thermal approaches based on the accumulation of degree days associated with phenological stages with variations in altitude, temperature, and precipitation [3,9], to more complex ecophysiological schemes that integrate the dynamics of carbon reserves and competition between vegetative and reproductive structures and the influence of thermal and water stress [23,24,25,26].

The objective of this study was to characterize the phenological stages of flower development and their relationship with climatic variables in ‘Hass’ avocados grown under tropical conditions at different altitudes. Understanding the environmental factors that regulate the flowering and phenological synchrony under these conditions provides a scientific basis for optimizing agronomic management practices and directing the harvest calendar for the ‘Hass’ cultivar in tropical systems.

## 2. Results

### 2.1. Altitudinal Climate Structure and Its Phenological Implications

**Figure 1:**
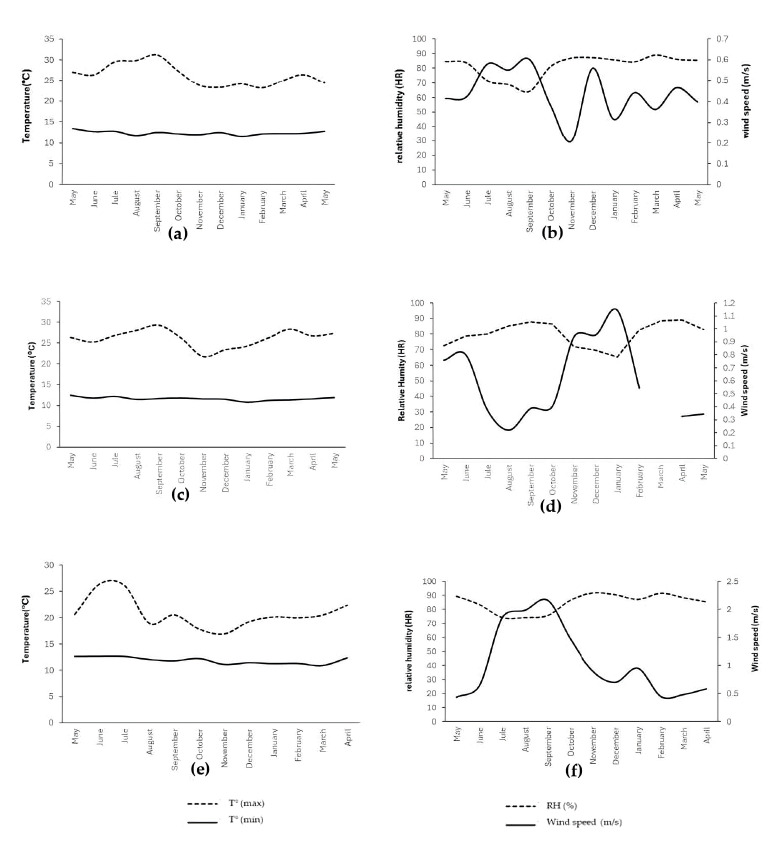
Climatic variables in the experimental plots. (**a**) Plot 3: maximum and minimum temperature; (**b**) Plot 3: relative humidity and wind speed; (**c**) Plot 5: maximum and minimum temperature; (**d**) Plot 5: relative humidity and wind speed; (**e**) Plot 23: maximum and minimum temperature; (**f**) Plot 23: relative humidity and wind speed. Note: (**d**) During the month of March, there was a malfunction in the wind speed meter.

The descriptive analysis (Table S1) showed an inverse thermal gradient among plots (L3, L5, and L23): 16.9 °C (L3), 15.3 °C (L5), and 14.7 °C (L23) (Figure 1). The climatic characterization revealed a clear and meaningful altitudinal gradient among the plots, establishing the environmental foundation upon which avocado flowering develops. The differences in temperature, humidity, and wind speed (Figure 1) represent distinct energetic and evaporative regimes that shape the microenvironment experienced by developing buds and inflorescences. The warmer and less humid conditions at L3, combined with lower wind exposure (Figure 1a,b), indicate an environment with reduced evaporative demand but greater thermal accumulation, which typically accelerates phenological processes. In contrast, the cooler, more humid, and more ventilated conditions at L23 (Figure 1e,f) suggest a microclimate where lower thermal availability and higher evaporative demand may constrain or delay floral developmental transitions.

The correlation structure reinforces this environmental architecture and highlights the interdependence among the climatic factors that shape phenological behavior (Table S2). The negative associations between mean temperature and either relative humidity (r = −0.615; *p* < 0.001) or rainfall (r = −0.199; *p* < 0.001), together with the positive correlation between wind speed and temperature (r = 0.214; *p* < 0.001) and the strong negative relationship between wind and humidity (r = −0.575; *p* < 0.001), reveal an atmosphere that alternates between warm, dry, and well-ventilated periods and cooler, more humid conditions. This oscillatory pattern defines the physiological context in which floral tissues must regulate hydration, turgor, and metabolic activity. Accordingly, these correlations delineate the climatic axes that ultimately explain the stage-specific phenological responses described later in the results.

The exploratory factor analysis (EFA) not only reduced the complexity of the climatic dataset but also revealed a coherent environmental structure underlying the phenological differences observed among sites. Factor 1 (21.9% of the variance) consistently integrated the thermal signal—mean and maximum temperature—and identified an inverse gradient with humidity and precipitation (Table 1), capturing a “heat–dryness” axis characteristic of warmer and less humid episodes. Factor 2 (20.0%) grouped wind speed and its negative relationship with humidity, representing a local ventilation pattern that influences evaporation and tissue water status. Factor 3 (12.3%) reflected the temporal structure of the climatic series—month and year—indicating that part of the variability corresponds to seasonal progression and interannual differences. Finally, Factor 4 combined minimum temperature and minimum wind speed, pointing to nighttime or low-turbulence conditions that may also affect thermal accumulation and energy balance (Table 2).

The relevance of this factorial architecture was supported by a significant Bartlett’s sphericity test (χ^2^ = 11,898; df = 45; *p* < 0.001) and a KMO of 0.556, appropriate for descriptive synthesis (Table S3). Confirmatory factor analysis (CFA) further reinforced this climatic structure, showing a robust model fit (CFI = 0.974; RMSEA = 0.163) (Table 3), and consolidating three principal atmospheric axes: temperature, wind, and humidity.

Together, these results demonstrate that climatic variability across the landscape is not random but structured into well-defined altitudinal gradients that determine thermal availability, ventilation, and local moisture conditions. ANOVA confirmed significant differences among plots for all climatic variables, with the lower site exhibiting warmer and drier conditions and the upper site characterized by cooler and more humid environments, alongside higher wind exposure typical of elevated terrain. The magnitude and significance of the F (251.4/117.6/37.7) and *p* (<0.001) values reinforce the robustness of this environmental stratification, which provides the foundation for understanding why avocado phenophases exhibit distinct and stage-specific sensitivities across the altitudinal gradient (Table S10). Methodologically, the combination of confirmatory factor analysis, principal component analysis, and ANOVA demonstrate the robustness of the multivariate approach for discriminating production environments. This approach enables the integration of meteorological information into phenological response models, improving the predictive capacity of local agroclimatic systems.

### 2.2. Phenological Dynamics of ‘Hass’ Avocado at Different Altitudes

Relevant phenological patterns were observed across the analyzed stages (EF010–EF719) along the altitudinal gradient (≈2056, 2212, and 2338 m.a.s.l.) over two consecutive productive cycles. The greatest deviations occurred during floral morphogenesis (EF110–EF115) and fruit development (EF610–EF617) (Tables S4, S5 and S9), indicating that these intermediate stages are particularly influenced by microenvironmental conditions and local climatic fluctuations. In contrast, the early phases of floral induction and differentiation exhibited lower relative variability, suggesting tighter regulation by cumulative thermal cues and a reduced influence of environmental stochasticity at the beginning of the reproductive cycle.

Coherent groupings among phenological variables representing successive transitions of the reproductive process were identified through principal component analysis (PCA) (Table 4). Bartlett’s test of sphericity confirmed the suitability of the dataset for factor reduction (χ^2^ = 19,200; *p* < 0.001), and the eigenvalue > 1 criterion allowed the retention of five significant components, demonstrating that avocado phenology does not respond to each stage in isolation, but is organized into interdependent developmental blocks. This multivariate structure suggests that the different phenological states are integrated within a continuous physiological progression, shaped by environmental gradients and internal regulatory mechanisms that manifest in an articulated manner throughout the cycle.

The structure derived from the PCA revealed a coherent sequence of phenological transitions consistent with the expected progression of the avocado reproductive cycle. The bud dormancy state (EF010), which showed negative loadings on the first three components, was clearly positioned at the opposite end of the axes associated with floral development and the initial stages of fruit formation, reflecting its role as a quiescent meristematic phase prior to reproductive activation. In contrast, one of the main components (PC2) grouped the intermediate stages of floral differentiation and inflorescence development (EF513–EF518), indicating that these phases share a common physiological basis characterized by active organogenesis and increasing metabolic demand. Another component integrated EF511–EF513, corresponding to the initial emergence of floral structures, while a third component clustered EF119, EF610, EF617, and EF715, representing the final steps of floral expansion, the transition toward anthesis, and the onset of early fruit set. Additional components isolated the early differentiation phases (EF019–EF115) and the vegetative flush together with initial fruit development (EF711–EF712), reflecting the natural alternation between vegetative and reproductive growth typical of the species.

The confirmatory factor analysis (CFA) supported the phenological structure identified in the PCA by validating four factors that represent clearly differentiated modules: (1) bud dormancy, (2) floral development, (3) floral morphogenesis, and (4) anthesis (Table 5). The correspondence between both analytical approaches indicates that the observed transitions are not isolated events but rather integrated stages within a continuous reproductive developmental pathway. The significant and negative covariances between vegetative and reproductive axes reflect the functional exclusivity between these processes, which is physiologically coherent: when the plant allocates resources to floral differentiation or reproductive expansion, vegetative activity decreases, and vice versa. The model fit indices showed outstanding performance (CFI = 0.976; TLI = 0.965; RMSEA = 0.043; 90% CI [0.0391; 0.0469]) (Table 6), confirming that the factorial solution robustly captures the variability of the recorded phenological states. This level of fit provides strong interpretative support for the model and demonstrates that avocado phenology can be explained through a reduced set of latent factors, facilitating its integration with environmental gradients and enabling progress toward more precise and biologically coherent predictive systems aligned with the species’ reproductive dynamics.

The graphical representations of the factors showed that the phenological progression of ‘Hass’ avocado follows a sequence clearly modulated by altitudinal microclimatic gradients, which determine the speed, synchrony, and efficiency of reproductive processes. The Welch ANOVA results revealed highly significant differences (*p* < 0.001) in the three main phenological dimensions and in bud dormancy (EF010), confirming that each phenological module responds differently to local environmental conditions. Component 3 (Anthesis), which showed the highest F value (F = 200.9; df1 = 2; df2 = 3688; *p* < 0.001), emerged as the phase most sensitive to thermal and hydric contrasts among altitudes, consistent with the strong microclimatic dependence of floral opening. This was followed by Component 1 (Floral morphogenesis; F = 159.2) and Component 2 (Floral development; F = 44.0), indicating that both induction and intermediate floral growth phases also respond markedly to environmental differences among sites. Bud dormancy (EF010), with the highest F value of the entire set (F = 305.4; *p* < 0.001), confirmed that the activation of the reproductive meristem is the process most tightly regulated by mean temperature and ambient humidity (Table S6). Together, these findings demonstrate that the microenvironment associated with altitude decisively regulates phenological expression, with anthesis and floral induction being the processes most influenced by the mountain thermal environment.

All stages of floral development, from morphogenetic induction (EF010) to anthesis (Component 3), showed highly significant differences between cycles (*p* < 0.001) (Table S7). The reduction in dormancy activity in the second cycle (mean difference = −10.7; *p* < 0.001) suggests a physiological adjustment potentially related to previous depletion of carbohydrate reserves. In contrast, the increase observed in floral morphogenesis (Component 1; difference = 0.435; *p* < 0.001) indicates a phenological recovery response, possibly associated with a rebalancing of resource allocation. The lower advancement of floral development in the second cycle (Component 2; difference = −0.402; *p* < 0.001) suggests that interannual climatic variations modified the expansion of reproductive structures, slowing the process. Finally, the increase in anthesis during the second cycle (Component 3; difference = 0.285; *p* < 0.001) reveals greater intensity and synchrony of floral opening, a pattern typical of compensatory phenological adjustment, integrating interannual climatic signals to modulate the reproductive strategy and maintain the functional dynamics of the alternant bearing pattern characteristic of this species.

### 2.3. Persistence and Modulation of Andean Thermal Seasonality

The 20-year record from the NASA station, located at 2600 m.a.s.l., showed a clear and persistent annual thermal cycle. Maximum (Tmax), mean (Tmean), and minimum (Tmin) temperatures exhibited marked monthly oscillations, confirming significant thermal seasonality even in a tropical context. Among these variables, Tmin presented the greatest amplitude of variation, with pronounced decreases between June and September, suggesting that nocturnal cooling is a key determinant of thermal seasonality in tropical high Andean zones.

**Figure 2:**
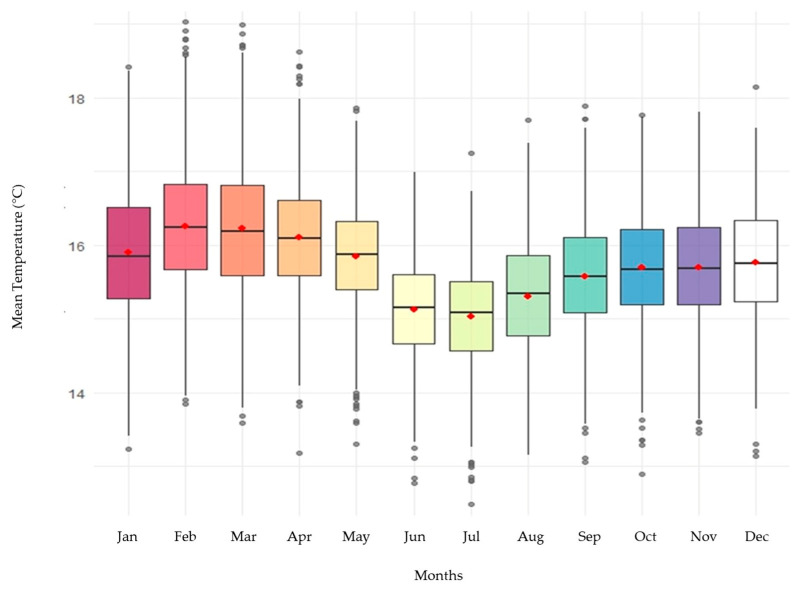
Distribution of monthly temperatures in box plots NASA station.

The distribution of monthly temperatures in box plots (Figure 2) showed clear differences among months, especially for Tmin, with lower values during the middle of the year. These patterns were statistically validated using one-way Welch’s ANOVA, which revealed significant differences among months for all three variables (Tmax: F = 57.2; Tmin: F = 307.9; Tmean: F = 197.2; *p* < 0.001). The Tukey post hoc test (Table S11) confirmed that June–August was significantly colder than the rest of the year, reinforcing the presence of a cold annual phase typical of tropical montane climates with bimodal rainfall regimes.

The seasonal component showed a stable intra-annual oscillation (Figure S1), whereas the trend component evidenced a gradual increase in mean temperature after 2010, consistent with warming reported for other high Andean regions of South America. The residual component represented short-term fluctuations not explained by seasonality, likely associated with transient meteorological events or synoptic variability. These results reflect a high persistence of the annual thermal cycle with a regular structure over time despite interannual variability.

The autocorrelation function (ACF) showed a significant positive peak at a 12-month lag, indicating that thermal conditions tend to repeat annually. The alternation of positive and negative correlations along the lag axis reflects a regular succession of warm and cold months, characteristic of a well-defined 12-month thermal cycle (Figure S2).

The progressive decrease in correlation amplitude at longer lags suggests a stationary series with persistent annual periodicity. This behavior confirms the stability of thermal dynamics in the high Andes, where variations depend more on solar radiation, cloud cover, and regional atmospheric circulation patterns than on abrupt continental or oceanic gradients. The predictability of this behavior confirms that the climatic records analyzed at 2466 m.a.s.l. can be taken as an appropriate reference for agroclimatic modeling based on thermal time (GDD), essential for agricultural phenology studies.

Monthly thermal amplitude, calculated as the difference between monthly means of daily maximum and minimum temperatures, averaged 1.22 °C across the 20 years. Although low relative to temperate regions, this is characteristic of equatorial montane systems, where high humidity and frequent cloudiness damp diurnal thermal extremes.

When stratifying the data by ENSO phases, amplitude decreased during El Niño (1.08 °C) and La Niña (1.01 °C) years compared with neutral years (1.40 °C). This indicates that both extremes tend to reduce daily thermal variation, likely due to increased cloudiness and changes in the radiative balance. During El Niño episodes, greater atmospheric stability and lower convection reduce nocturnal cooling; during La Niña years, increased cloudiness and rainfall also attenuate amplitude. These results demonstrate the sensitivity of the high Andean thermal regime to ocean–atmosphere oscillations and the need to consider ENSO phase in phenological and productivity models.

In contrast, the 10-year record from the La Nubia station (IDEAM, 2100 m.a.s.l.) did not show a defined seasonal thermal structure. Monthly averages of Tmax, Tmin, and Tmean remained homogeneous throughout the year, without a systematic repetition of warm or cold phases. Although ANOVA indicated significant differences among months, these did not correspond to a cyclical annual pattern but rather to random interannual fluctuations. STL decomposition confirmed this absence of structure: the seasonal component was weak or nonexistent, and variability was dominated by the residual component. Mean thermal amplitude (0.88 °C) was lower than at 2600 m, with unclear differences among ENSO phases (Neutral = 0.91 °C; El Niño = 1.28 °C; La Niña = n.d.). This suggests that at lower altitudes, temperature responds more to local microclimatic and mesoscale processes than to regional or global climatic forcings.

This altitudinal difference shows that higher zones (≥2500 m) exhibit a more marked and predictable annual thermal oscillation, whereas mid-elevation zones (~2100 m) show more stable and stochastic conditions, with direct implications for phenological synchrony and management of perennial, climate-sensitive crops. The confirmation of a well-defined annual thermal periodicity at 2600 m.a.s.l. provides a solid basis for calibrating phenological models dependent on thermal time or growing degree days. The regularity of the thermal cycle allows more precise predictions of biological events such as flowering, fruit set, and ripening in high-elevation crops, provided that base and upper thermal thresholds are known.

### 2.4. Thermal Synchrony and Phenological Stability in Altitudinal Gradients

**Figure 3:**
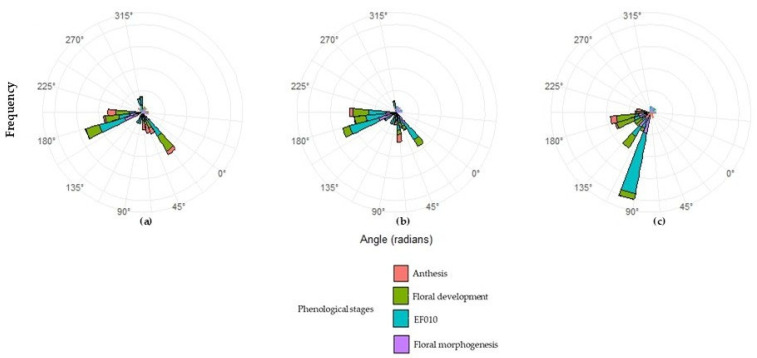
Circular analysis of phenological synchrony for the plots evaluated. (**a**) Plot 3, (**b**) Plot 5, (**c**) Plot 23.

Circular analysis applied to thermal accumulation (GDD) identified precise patterns in the occurrence of main phenological states of ‘Hass’ avocado, expressed in angular terms and accompanied by the concentration coefficient (ρ) (Table 7; Figure 3). This parameter quantifies temporal synchrony: values of ρ close to 1 represent high concentration and thus greater homogeneity among individuals, whereas low values indicate wide temporal dispersion. The results show marked differences between L3, L5, and L23, reflecting the influence of altitude and microclimatic conditions on reproductive phenology. In general, ρ ranged from 0.12 to 0.82, indicating that later phenological events tend to be more synchronized than initial states. This trend suggests progressive thermal coherence: as the cycle advances, plants respond more uniformly to heat accumulation.

Circular analysis revealed that phenological synchrony in ‘Hass’ avocado can be modulated by the altitudinal gradient, which determines the coherence and stability of each phase of the reproductive cycle (Table 7; Figure 3). At the lowest-altitude site (L3), the wide dispersion observed in the early stages (ρ = 0.13 in EF010) indicates a greater influence of local microclimate and lower cohesion in the activation of the reproductive meristem. However, the high synchrony observed during anthesis (ρ = 0.77) suggests that once the required thermal thresholds are reached, reproductive processes converge physiologically. In the intermediate zone (L5), phenological progression exhibited greater thermal stability and synchrony (ρ = 0.82 in anthesis), indicating that these altitudinal conditions favor a more uniform reproductive response. At the highest altitude (L23), phenological states shifted toward later angular values, reflecting a more prolonged cycle; even so, dormancy was notably homogeneous (ρ = 0.63), while floral morphogenesis showed greater dispersion (ρ = 0.17), indicating particular sensitivity to cold and variable conditions. Overall, the altitudinal pattern indicates that intermediate elevations (~2200 m) generate the highest phenological synchrony, whereas lower or higher elevations show greater variability in early-phase responses.

Circular analysis based on day of the year (DOY) complemented the thermal interpretation by situating phenological events within their calendar context (Table S8). Across the three sites, bud dormancy (EF010) and floral morphogenesis concentrated between days 90 and 180, while floral development and anthesis occurred between days 150 and 195. Concentration values (ρ = 0.24–0.94) showed higher phenological stability at L23 (ρ > 0.8) and greater dispersion at L3 and L5 (ρ < 0.4), confirming that daily thermal amplitude is a key driver of temporal variability. This pattern suggests that colder altitudes reduce microclimatic variability, enabling a more stable phenological progression.

Comparison between GDD- and DOY-based analyses revealed a robust physiological correspondence between thermal accumulation and phenological occurrence. Angular values and mean calendar dates remained consistent across methods, confirming that accumulated temperature is a reliable predictor of reproductive advancement in avocado. This behavior reflects a clear thermal control over floral induction and a final synchronization of flowering associated with thermal and hormonal signals that stabilize anthesis and ensure reproductive efficiency in montane environments.

Taken together, the results demonstrate that the combined use of circular statistics and GDD is a powerful tool for characterizing phenological dynamics in tropical systems. This approach allows quantification of synchrony, detection of subtle thermal shifts across altitudes or years, and, from an agroclimatic perspective, the identification of fundamental principles: (i) early phases are more sensitive to thermal variability; (ii) reproductive phases respond to defined thermal thresholds; and (iii) greater synchrony at 2200–2300 m indicates an optimal altitudinal range for productive stability. Methodologically, angular representation surpasses the limitations of traditional chronological time, enabling comparisons between non-consecutive years and supporting projections under climate-change scenarios.

## 3. Discussion

The climatic structure observed along the altitudinal gradient revealed not only a physical stratification of temperature, humidity, and wind exposure, but also a set of microenvironmental conditions with clear physiological implications for the phenology of ‘Hass’ avocado. These conditions mirror hydroclimatic patterns of the Tropical Andes, where altitude modulates atmospheric moisture, radiative balance, and ventilation [27,28]. Such gradients create distinct evapotranspirative environments that regulate hydration, turgor, and carbohydrate dynamics—processes that fundamentally support floral induction and reproductive development. Confirmatory factor analysis validated temperature, wind, and humidity as the dominant environmental axes structuring the production environment, consistent with other tropical montane systems where thermal availability governs developmental rate, wind modulates evaporative demand, and humidity stabilizes tissue water status [28].

These altitude-driven climatic contrasts exerted a strong influence on the timing and stability of reproductive development. At lower elevations, greater thermal availability accelerated bud dormancy release and advanced reproductive initiation, whereas colder and more humid conditions at higher elevations delayed these transitions, as reported for orchards in Mexico [10,29]. The significant differences observed among elevation bands also align with reports from tropical perennial systems showing that altitudinal gradients create contrasting microclimates that modulate developmental rates [30,31]. Wind exposure at higher altitudes likely increased evaporative stress on reproductive tissues, while greater humidity buffered desiccation risk but increased pathogen pressure—factors shown to affect flowering, pollen viability, and the synchrony of male and female phases in Chilean and Mexican orchards [29,32,33].

Taken together, these findings demonstrate that the phenological responses of ‘Hass’ avocado are governed by an integrated environmental architecture defined by altitude-driven gradients in temperature, wind, and humidity. Such gradients determine the physiological conditions under which floral induction, morphogenesis, anthesis, and initial fruit development occur. This environmental–physiological coupling is consistent with recent studies highlighting the strong phenological plasticity of avocado in regions with pronounced topographic variation [10,34]. Methodologically, the combined use of EFA, CFA, and ANOVA provided a robust multivariate framework for characterizing phenological modules and integrating meteorological information into phenological response models, supporting previous applications in tropical fruit-tree zonification and modeling efforts [28,35].

The phenological progression across stages EF010–EF719 confirmed a structured transition from bud dormancy to anthesis and fruit development, consistent with the BBCH scale for avocado. Anthesis (Component 3) emerged as the most environmentally sensitive stage, confirming earlier findings that low temperatures extend the floral cycle, restrict pollen tube growth, and reduce fertilization potential [36,37]. The stronger morphogenetic activity and more intense anthesis observed in the second cycle agree with Lovatt’s [38,39] findings that floral differentiation is governed by the carbon–nitrogen balance during induction. The ON–OFF alternation observed here corresponds with physiological mechanisms described by Lovatt and Salazar-García [40], where developing fruits act as dominant sinks in “ON” years, reducing carbohydrate availability for subsequent inflorescence differentiation. Recent evidence from high-altitude Colombian orchards [19] similarly reports pronounced alternation in flowering intensity, fruit load, and vegetative flush driven by climate variability and sink competition, reinforcing the physiological basis of our observed interannual variation.

Thermal seasonality at 2600 m.a.s.l. displayed predictable annual temperature cycles linked to the solar cycle and ENSO-related anomalies [41,42], while strong 12-month autocorrelation supported the use of growing degree days (GDD) as a reliable phenological predictor [9]. Circular analysis revealed that thermal synchrony (ρ) increased from early phenophases to anthesis, indicating progressive homogenization as heat accumulates. These patterns match observations from Colombian highlands [9] and show that altitude reorganizes the internal temporal structure of the reproductive cycle: early-stage dispersion at low altitude (L3), homogeneous dormancy at high altitude (L23), and maximum synchrony at intermediate altitude (L5). The congruence between GDD- and DOY-based circular metrics confirms that accumulated temperature is a central regulator of reproductive progression. Mechanistically, the strong thermal-driven synchronization of anthesis is consistent with experimental work in California showing that sequential cool–warm conditions activate coordinated expression of flowering-time and floral organ identity genes [14]. Broader spatial consistency comes from Chile, where flowering is highly sensitive to temperature fluctuations, with warmer conditions enhancing flower opening and pollinator activity and colder spells reducing the effective flowering window [32].

Phenological synchrony has strong agronomic implications. At intermediate altitudes (~2200–2300 m), high synchrony promotes more uniform fruit maturation, which concentrates harvest windows, improves packhouse efficiency, reduces labor fluctuations, and increases predictability for export markets [10,43]. Conversely, asynchronous flowering at extreme altitudes generates non-uniform maturity, extended harvest periods, variable quality, and unpredictable export flows—issues documented in Chilean orchards [32]. Although this study focuses on ‘Hass’, similar altitude–temperature interactions may occur in other cultivars with distinct thermal requirements, such as ‘Fuerte’ and ‘Reed’ [36,37], and in other high-elevation perennials like coffee [44], suggesting that altitude-driven phenological structuring is a general feature of montane crops. Beyond their ecological relevance, these patterns have direct implications for orchard management. Higher synchrony at intermediate altitudes enables more accurate scheduling of pruning, fertilization, and harvest operations, while also improving the planning of labor and postharvest logistics. Conversely, reduced synchrony at extreme elevations signals the need for differentiated management calendars and more flexible harvest programs to maintain fruit quality and market consistency.

At the physiological level, the altitudinal and interannual patterns reported here correspond to well-established mechanisms. Carbohydrate partitioning governs alternate bearing [38,40], while thermal thresholds regulate floral induction [36]. Hormonal regulation—particularly involving gibberellins, cytokinins, and ABA—modulates reproductive meristem activity, resource allocation, and pollen viability [39]. Microclimatic stressors such as cold nights, humidity, and wind can also reduce pollen tube growth, explaining the sensitivity of floral morphogenesis and anthesis to local temperature fluctuations [29,33].

In a recent study on the impacts of climate change on horticultural systems, Fanou-rakis et al. [45] highlight that rising temperatures, increased vapor-pressure deficit, and greater water stress are emerging as the main threats to productivity and phenological stability across multiple crops. Their findings reinforce the likelihood that the optimal synchrony band identified in this study (~2200 m) may shift to higher elevations as warm-ing intensifies, increasing the need for climate-responsive orchard design, improved wa-ter-management strategies, and the selection of genotypes with enhanced thermal resili-ence. Taken together, these elements underscore the importance of thermal–circular pre-dictive frameworks as essential tools for climate-adaptive planning in high-Andean avo-cado production.

Finally, although the absence of orchards between 1600 and 2000 m represents a limitation that constrains full characterization of the altitudinal response curve, the integration of circular statistics, thermal accumulation, and multivariate analysis provides a robust foundation for agroclimatic zoning, orchard site selection, harvest scheduling, and climate-change adaptation. Overall, this study demonstrates that altitude modulates ‘Hass’ avocado phenology through tightly coupled thermal and hydric mechanisms and that optimal biological and operational synchrony occurs at intermediate elevations.

## 4. Materials and Methods

### 4.1. Location and Characteristics of the Study Area

The study was conducted in the municipality of Pijao, Quindío Department, Colombia, located on the western flank of the Central Andean Mountain Range (4°19′ N, 75°43′ W). The study area lies within a zone of medium to steep Andean slopes, with an altitudinal gradient ranging from 2070 to 2638 m.a.s.l. [46]. The soils are derived from volcanic materials and classified as Andisols, characterized by high porosity, good drainage, and intermediate moisture retention capacity—typical features of soils in Colombia’s coffee-growing region [47]. Land use is dominated by agroforestry systems and perennial crops, particularly coffee (*Coffea arabica*), forestry plantations, and, more recently, avocado (*Persea americana* Mill cv. ‘Hass’).

The region’s climate is classified as humid temperate mountain, characterized by a bimodal rainfall pattern with two distinct rainy seasons (April–May and October–November) and two relatively dry seasons (January–February and July–August) [48]. The mean annual temperature ranges from 17.5 to 28 °C, varying significantly based on the morphology and altitude of the mountains [49].

Altitudinal and geographical variations in the study area, combined with the climatic particularities observed during the experimental period, resulted in high microclimatic heterogeneity, directly influencing the spatial and temporal variability of the analyzed meteorological variables. To evaluate the effect of the altitudinal gradient, three experimental plots representative of different elevations were selected, previously identified by the producer as the lower, intermediate, and upper sections of the plantation:

• Plot 1 (L1), corresponding to the lowest elevation site, with trees located between 2070 and 2080 m.a.s.l.
• Plot 2 (L2), representing the intermediate elevation, ranging from 2293 to 2303 m.a.s.l.
• Plot 3 (L3), the highest site, with trees situated between 2625 and 2685 m.a.s.l.

Geographic coordinates and elevations were determined using a Garmin eTrex 32X (GPS receiver, Garmin, Olathe, KS, USA) with a measurement error of ±10 m. Georeferencing was performed ensuring the highest possible precision of the sampling points. Regional and Historical Climate Characterization.

### 4.2. Historical Climate Characterization

To contextualize the climate conditions of the study area, records from two reference meteorological stations located at different altitudes were used. The first source was the La Nubia station (IDEAM), located at 2104 m.a.s.l. (5°02′58″ N, −75°28′11″ W), with daily records for the period 2012–2022. The second source was the NASA/POWER (Prediction of Worldwide Energy Resources) satellite database, spatially adjusted to the coordinates 4.26° N and −75.56° W, with an average altitude of 2466 m.a.s.l., which provided data for the period 2020–2024. Both sources were used exclusively to characterize the historical and regional climatic behavior of the area, considering the variables maximum temperature (Tmax), minimum temperature (Tmin), mean temperature (Tmean), relative humidity (RH), precipitation, and wind speed. From the daily series, monthly and annual mean values were calculated, as well as the daily thermal amplitude (ATd = Tmax − Tmin). Intra-annual variation was represented through the monthly (ATm) and annual (ATa) thermal amplitudes, which were used as indicators of local thermal stability.

Thermal seasonality was evaluated through the additive decomposition of the monthly mean temperature series, to separate the observed signal into structural components that characterize processes operating at different temporal scales. The original series Tt was represented under the model (Equation (1)):

(1) T_t_ = T_tremd_ + T_seasonal_ + T_residual_

where Tₜ corresponds to the observed monthly mean temperature, Ttrend is the secular or long-term component, Tseasonal describes the recurrent annual thermal cycle, and Tresidual contains the variation not explained by the preceding components.

The long-term trend was obtained using a centered 12-month moving-average smoothing method, which reduces intra-annual variability and highlights multi-year patterns in the thermal signal (Equation (2)).

(2) $$T_{tremd}=MA_{12}(T_{t})$$

where MA_12_ represents the centered moving average with a one-year window.

The annual seasonal cycle was derived by grouping the residual values (observed series minus trend) by month of the year and calculating, for each month, the mean across all available years. Thus, for each month *m*, the seasonal component was estimated as (Equation (3)):

(3) $$T_{seasonal}(m)=\frac{1}{N}\sum_{i=1}^{N}\left(T_{t(m,i)}-T_{tremd}(t(m,i))\right)$$

where *m* is the month of the year (1–12), *i* indexes the years, *N* is the total number of available years, *t*(*m*,*i*) identifies the position in the series corresponding to month m in year *i*.

This procedure yields a seasonal cycle composed of twelve values that capture the recurrent thermal structure characteristic of the local climate.

The residual component was obtained by Equation (4):

(4) $$T_{\mathrm{residual}}=T_{t}-(T_{tremd}+T_{\mathrm{seasonal}})$$

This equation captures the high-frequency variability associated with non-periodic meteorological events, monthly anomalies, instrumental noise, or variations not explained by the deterministic components.

### 4.3. Recording Experimental Meteorological Data

During the study period, three automatic meteorological stations were installed in experimental plots representative of the study area, located at different altitudes: Plot 3 (2070 m.a.s.l), Plot 5 (2303 m.a.s.l), and Plot 23 (2600 m.a.s.l). The stations consisted of (Davis Vantage Pro2, USA) equipment, equipped with air temperature, relative humidity, rain gauge, and anemometer sensors, connected to a data logger with backup and cloud synchronization. Records were collected at hourly intervals and aggregated into daily series for analysis. Daily averages of temperature and humidity, daily maxima and minima, total precipitation, and mean wind speed were calculated. All series were expressed in the local time zone (UTC−5). The stations were installed within the altitudinal range corresponding to each plot. In addition, the site selection followed the criteria established in the equipment’s technical manual, which included: having an open area of at least 15 m, ensuring a leveled surface for sensor installation, and delimiting the area to prevent access by unauthorized persons.

Quality control included: (i) verification of temporal continuity and detection of missing data, (ii) identification of outliers using statistical rules and physical limits, (iii) cross-verification among variables, and (iv). Short gaps (<48 h) were filled by linear interpolation, whereas longer intervals were retained as missing data and treated according to the requirements of each analysis.

### 4.4. Phenological Evaluation of the Crop

The phenological monitoring was carried out on six–year–old adult ‘Hass’ avocado trees. Sexual seedlings of the same variety were used as rootstock. The trees were in three experimental plots distributed along the altitudinal gradient. In each plot, 30 representative trees were selected based on homogeneous morphology, comparable vegetative vigor, and a uniform fruit load, to reduce intrapopulation variability. Trees showing disease symptoms or poor floral development were excluded. On each tree, four healthy and structurally similar branches were marked, located in the middle third of the canopy and oriented toward the four cardinal directions (N, S, E, and W). On each branch, a one–meter segment was delimited, measured from the apex toward the base of the stem. These segments constituted experimental units for phenological monitoring.

A uniform agronomic management was applied across the three experimental plots. Soil fertilization was carried out using macronutrients (N, P, and K) and micronutrients according to soil nutrient availability. Additionally, foliar applications of boron and zinc were performed prior to floral development. None of the plots had irrigation systems, so water availability depended exclusively on rainfall. Phytosanitary management followed an Integrated Pest Management (IPM) approach, using biological and synthetic chemical products selected based on their efficacy and compatibility with the crop.

At the beginning of the monitoring period, the total number of buds present on each of the previously marked branches was counted, recording their initial phenological stage. From that point onward, weekly evaluations were conducted to identify and record visible changes in the developmental stage of each bud, following the BBCH phenological scale adapted for avocado [12].

Each bud was classified into one of the macro-stages EF0, EF5, and EF6, corresponding to the phases of budburst, floral morphogenesis, and flowering, respectively, including their specific substages (Table 8). These stages sequentially describe the formation and development of inflorescences, from initial dormancy to flower opening, allowing the entire flowering process to be captured. Likewise, buds that did not complete their development due to death, damage, or loss of tracking were recorded and considered as missing data.

The phenological stages were recorded using standardized digital forms and converted to percentages relative to the initial number of buds (EF010). This approach enabled a more precise and dynamic monitoring of phenological transitions, allowing for the comparison of the evolution of reproductive structures among trees and experimental plots. Buds, inflorescences, or shoots that became diseased or disappeared during the study were considered missing data.

### 4.5. Data Analysis

#### 4.5.1. Experimental Meteorological Data

The statistical analyses were done in three phases: Phase I. Exploration and preparation of climatic data; Phase II. Identification of latent structures through Principal Component Analysis (PCA) and Exploratory Factor Analysis (EFA); and Phase III. Validation of the structure through Confirmatory Factor Analysis (CFA) and comparison among plots.

(a) Phase I: Environmental temperature series (mean, maximum, and minimum, °C), relative humidity (RH), precipitation (mm), and wind speed (mean and maximum, m·s^−1^) were evaluated. The precipitation variable was transformed using log (x + 1) to reduce skewness, and relative humidity was expressed as a proportion (0–1) to improve comparability among variables. Descriptive statistics and correlations were computed as input for subsequent multivariate analyses.
(b) Phase II: Principal Component Analysis (PCA) was applied as an exploration technique to reduce dimensionality and detect natural groupings among climatic variables. Subsequently, an Exploratory Factor Analysis (EFA) was conducted using the minimum residual extraction method with oblique rotation (oblimin), assuming correlation among factors. The Kaiser criterion (eigenvalues > 1) and parallel analysis determined the number of factors retained.
(c) Phase III: Based on the PCA and EFA results, a three-factor structure with coherent physical interpretation was defined:
  - Temperature, integrating thermal variables (mean, maximum, and minimum temperature).
  - Wind, comprising mean and maximum wind speed.
  - Humidity, represented by relative humidity.

Confirmatory Factor Analysis (CFA) was applied to validate the latent climatic structure across the three experimental plots. Parameters were estimated to be using robust maximum likelihood (MLR), considering the treatment of missing data through Full Information Maximum Likelihood (FIML). Model fit quality was assessed using the indices CFI, TLI, SRMR, and RMSEA. Finally, factor scores were computed using the regression method and employed to compare plots through one-way ANOVA and Tukey’s post hoc tests (*p* ≤ 0.05), aiming to identify significant differences in climatic components among plots.

#### 4.5.2. Phenological Analysis of the Crop

The statistical analyses were structured into five complementary phases aimed at characterizing the phenological dynamics and evaluating the latent structure of the reproductive developmental stages.

Phase I. Data Exploration and Preparation: Weekly phenological observations of the marked buds on each tree were consolidated, expressing the relative counts of each phenological stage (PS) as a percentage of the initial number of latent buds (PS010). This standardization helped reduce the effect of variability among branches and trees, allowing direct comparison of phenological progression across experimental units. Subsequently, a descriptive exploration of the data was conducted, and Pearson correlations were calculated among phenological stages to identify the magnitude and direction of associations, as well as potential transitions between developmental phases.

Phase II. Identification of Latent Structures through Principal Component Analysis (PCA): To explore the underlying patterns of variation and reduce the dimensionality of the dataset, a Principal Component Analysis (PCA) was applied to the relative percentages of phenological stages corresponding to the BBCH macro-stages 0, 5, and 6, which represent the phases of budburst, floral morphogenesis, and flowering. Sampling adequacy for the factorial analysis was assessed using Bartlett’s test of sphericity and the Kaiser–Meyer–Olkin (KMO) index. The PCA was performed using the correlation matrix and the Varimax orthogonal rotation method to maximize component interpretability. Components with eigenvalues greater than 1 were retained, and variables with factor loadings equal to or greater than 0.40 were considered for biological interpretation. The extracted components were interpreted based on the phenological stages with the highest contributions, representing axes associated with the main phases of reproductive development.

Phase III. Structural validation through Confirmatory Factor Analysis (CFA): To evaluate the validity of the latent structure identified in the PCA, a Confirmatory Factor Analysis (CFA) was applied using the maximum likelihood estimation method. The theoretical model was constructed considering four latent factors representing the predominant phenological phases:

(1) Latency (EF010),
(2) floral morphogenesis (EF511–EF512),
(3) floral development (EF513–EF518), and
(4) anthesis (EF620–EF617).

The quality of the model fit was evaluated through goodness-of-fit indicators, including the Comparative Fit Index (CFI), the Tucker–Lewis Index (TLI), and the Root Mean Square Error of Approximation (RMSEA). The reference values to consider an adequate fit were established according to commonly accepted criteria in the literature: CFI and TLI greater than 0.95, and RMSEA lower than 0.05. The CFA allowed contrasting the congruence between the observed phenological structure and the proposed theoretical model, confirming the sequence of transitions among floral morphogenesis, floral development, and anthesis (full flowering).

Phase IV. Graphical representation and temporal relationship: Subsequently, the identified factors were graphically represented using bar charts, employing the sum of the relative percentages of each phenological stage instead of standardized values (Z-scores). This approach allowed expressing the cumulative contribution of the stages in terms of their relative frequency, providing a more direct and quantitatively interpretable visualization of phenological variations among plots. Additionally, accumulated thermal time was calculated using a base temperature of 10 °C (Equations (5) and (6)), based on daily temperature records, to express phenological changes as a function of thermal development [50]. This approach enabled the relationship between phenological transitions and the thermal conditions of each environment to be established, supporting a more precise physiological interpretation of the developmental process.

(5) $$\mathrm{GDD}=\sum_{i=1}^{n}\left(Tp-Tb\right)i$$

(6) If Tp>Tb↠GDD=Tp−Tb

where GDD corresponds to the Growing degree-days (°C), i represents days, Tp daily average temperature, and Tb the base temperature at which growth stops (10 °C) [50].

Phase V. Circular analysis: To describe the synchrony and temporal distribution of the main phenological events, a circular statistical analysis was applied using the phenological factors derived from the confirmatory factor analysis. This approach allowed the recurrent events of the reproductive cycle to be represented in an angular space, where each observation was associated with an angle reflecting its relative position within the annual or thermal cycle.

For this purpose, the values of the phenological stages were transformed into circular angles (θ) as a function of accumulated thermal time (degree-days, base 10 °C) and, complementarily, as a function of the day of the year (DOY), to assess the consistency between both temporal scales. In this way, phenological changes were interpreted in cyclic units of 360°, representing the annual or seasonal recurrence of reproductive development. From the angular distributions, mean vectors and their corresponding mean directions (μ) were estimated, indicating the average timing of occurrence of each phenological event. In addition, the concentration coefficient (ρ) was calculated, quantifying the degree of clustering of the data around the circular mean. The circular analysis was performed independently for each plot along the altitudinal gradient, allowing the identification of shifts in the mean timing of occurrence of phenological stages and variations in their concentration.

Graphical representations were generated through circular diagrams and mean vectors, which facilitated comparison among plots and interpretation of the temporal sequence of phenological events as a function of thermal accumulation. Data processing and analysis were performed in the R statistical environment (version 4.3.2) using the RStudio 2022.07.1 interface. The packages dplyr and tidyr were used for data cleaning and organization, lubridate for handling temporal variables, psych and facto-extra for exploratory factor and principal component analyses, lavaan for confirmatory factor analysis, CircStats and circular for circular statistics, and ggplot2 for graphical representation of the results.

## 5. Conclusions

This study provides compelling evidence that altitude-driven microclimatic gradients are a dominant force shaping the phenological behavior of ‘Hass’ avocado in the tropical Andes. By integrating multivariate (EFA, CFA, ANOVA) and circular–thermal approaches, we demonstrate that temperature, humidity, and wind define distinct environmental niches that regulate the timing and synchrony of floral induction, morphogenesis, and anthesis. The identification of an altitudinal optimum around ~2200 m.a.s.l.—where phenological synchrony, reproductive stability, and GDD-based predictability converge—offers a framework for improving orchard establishment, harvest planning, and market-oriented production strategies. These results strengthen the eco-physiological basis for agroclimatic zoning and provide actionable insights for enhancing packhouse efficiency, reducing labor variability, and ensuring supply consistency in export-oriented avocado chains. As mountain climates increasingly respond to warming trends, the demonstrated sensitivity of flowering synchrony to altitude underscores the relevance of these findings for climate-resilient management and long-term planning. Although conducted on a single cultivar and region, the findings provide a robust methodological platform for broader multi-regional and multi-cultivar studies aimed at improving phenological modeling and climate-adaptive management, and climate-informed decision-making in high-elevation avocado systems.

## Figures and Tables

**Table 1:** Result of exploratory factor analysis (EFA) of altitudinal climate structure and its phenological implications.

Factors	Loadings	Variance (%)	Cumulative (%)
1	2.192	21.92	21.9
2	2.001	20.01	41.9
3	1.226	12.26	54.2
4	0.772	7.72	61.9

**Table 2:** Result of exploratory factor analysis (EFA) of altitudinal climate structure and its phenological implications.

Identification	1 **	2 **	3 **	4 **
Log(x + 1) (rainfall)	−0.329			
(RH) *	−0.543	−0.353		
Average Ambient Temperature (°C)	0.959			
Maximum Ambient Temperature (°C)	0.845			
Minimum Ambient Temperature (°C)				0.610
Average Wind Speed (m/s)		0.995		
Maximum Wind Speed (m/s)		0.869		
Minimum Wind Speed (m/s)				0.431
Month			−0.391	
Year			0.998	

The ‘Minimum residue’ extraction method was used in combination with an ‘oblimin’ rotation. * (RH): Relative Humidity was expressed as a ratio from 0 to 1. ** number of factors yielded by the analysis.

**Table 3:** Result of Confirmatory validation (CFA) of altitudinal climate structure and its phenological implications.

CFI	TLI	SRMR	RMSEA
0.974	0.914	0.0330	0.163

**Table 4:** Result of principal component analysis (PCA) of phenological dynamics of ‘Hass’ avocado.

Phenological Stages			Component		
	1	2	3	4	5
EF010	−0.697	−0.573	−0.339		
EF019				0.539	
EF110				0.617	
EF115				0.542	
EF119			0.319		
EF510					
EF511		0.497			
EF512		0.777			
EF513	0.538	0.415			
EF514	0.571				
EF515	0.521				
EF517	0.408				
EF518	0.539				
EF610			0.515		
EF617			0.664		
EF711					0.727
EF712					0.609
EF715			0.511		
EF719					

The analysis used varimax rotation.

**Table 5:** Confirmatory factor analysis of phenological dynamics of ‘Hass’ avocado.

Factor	Indicator	Estimator	Standard Error	Z	*p*
1	EF010	41.29	0.3741	110.39	<0.001
2	EF513	9.06	0.2138	42.38	<0.001
	EF514	2.85	0.0934	30.48	<0.001
	EF515	1.80	0.0693	26.04	<0.001
	EF517	1.48	0.0577	25.59	<0.001
	EF518	3.70	0.1103	33.50	<0.001
3	EF511	1.05	0.0948	11.09	<0.001
	EF512	10.62	0.6301	16.86	<0.001
4	EF610	2.10	0.2240	9.40	<0.001
	EF617	4.14	0.4060	10.20	<0.001

Loads of the resulting factors.

**Table 6:** Result of Confirmatory validation (CFA) of phenological dynamics of ‘Hass’ avocado.

CFI	TLI	RMSEA	CI *	
			Lower	Upper
0.976	0.965	0.043	0.0391	0.0469

* Confidence interval 90% (CI).

**Table 7:** Circular analysis applied to thermal accumulation (GDD).

Plots	Stages	Average	(ρ)	Average Grades	Average GDD
L3	Anthesis	−51.75	0.77	−51.75	−109.97
	Floral development	−117.84	0.63	−117.84	−250.43
	EF010	−52.54	0.13	−52.54	−111.66
	Floral morphogenesis	−142.55	0.39	−142.55	−302.94
L5	Anthesis	−55.00	0.82	−55.00	−116.88
	Floral development	−89.41	0.59	−89.41	−190.01
	EF010	−94.43	0.40	−94.43	−200.67
	Floral morphogenesis	−117.97	0.57	−117.97	−250.69
L23	Anthesis	−56.05	0.66	−56.05	−119.12
	Floral development	−114.73	0.12	−114.73	−243.81
	EF010	22.52	0.63	22.52	47.86
	Floral morphogenesis	50.12	0.17	50.12	106.51

**Table 8:** BBCH phenological coding for ‘Hass’ avocado, macro–stages 0, 5, and 6.

Macro-Stages	Identification	Figure	Stages *
EF0	Buds	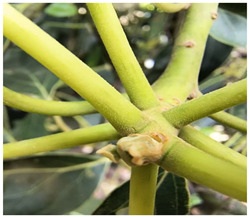	EF010 native buds: buds are closed and covered by scales.
EF1	Reproductive development	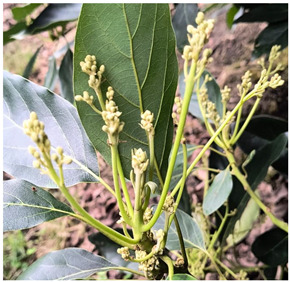	EF510 Inactive reproductive shoots: shoots covered by scales. EF511 Beginning of reproductive bud swelling: light brown bud scales start to separate. EF512 End of reproductive bud swelling: bud scales completely separated. 513 Reproductive bud burst: bud scales folded backward, inflorescences barely visible. EF514 Compound inflorescence separation: individual inflorescences separated, and elongation begins. EF515 Inflorescences at 50% of final length: secondary axes elongated, tertiary axes still covered by bracts, small, closed flowers. EF517 Inflorescences at 70% of final length: tertiary axes elongated, individual flowers separated.
EF5	Flowering	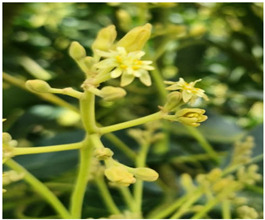	EF610 First flowers open. EF617 (70%) of flowers open.

* Based on the stages proposed by Alcaraz, 2013 for BBCH codification [12].

## Data Availability

The original contributions presented in this study are included in the article/Supplementary Materials. Further inquiries can be directed to the corresponding author.

## Supplementary Materials

The following supporting information can be downloaded at: https://www.mdpi.com/article/10.3390/plants14243822/s1, Table S1: Descriptive analysis of altitudinal climate structure and its phenological implications. Table S2: Correlation matrix of altitudinal climate structure and its phenological implications. Table S3: Bartlett Sphericity Test of altitudinal climate structure and its phenological implications. Table S4: Descriptive analysis of phenological dynamics of ‘Hass’ avocado. Table S5: Partial correlation of phenological dynamics of ‘Hass’ avocado. Table S6: Welch’s ANOVA of phenological dynamics of ‘Hass’ avocado. Table S7: Welch’s ANOVA and Tukey post hoc tests of phenological dynamics of ‘Hass’ avocado. Table S8: Circular analysis applied today of year (DOY). Table S9: Description of phenological stages. Table S10: Welch’s ANOVA of Altitudinal climate structure. Table S11: The Tukey post hoc test for temperature among months. Figure S1: Seasonal–Trend decomposition using Loess (STL). Figure S2: Autocorrelation function (ACF).

## Abbreviations

The following abbreviations are used in this manuscript:

Abbreviations	Meaning
EFA	Exploratory factor analysis
PCA	Principal component analysis
CFA	Confirmatory Factor Analysis
CFI	Comparative Fit Index
TLI	Tucker–Lewis Index
RMSEA	Root Mean Square Error of Approximation
SRMR	Standardized Root Mean Square Residual
ACF	Autocorrelation function
GDD	Growing degree days
DOY	Day of year
STL	Seasonal–Trend decomposition using Loess
ENSO	El Niño-Southern Oscillation
