# Supplementary material for: Climatic Drivers of Flowering Synchrony in ‘Hass’ Avocado Under Tropical Andean Conditions
Source: Plants (Basel). 2025 Dec 16;14(24):3822. doi: 10.3390/plants14243822 (PMC12737019; doi:10.3390/plants14243822)
Supplement: Supplementary file 1 [file plants-14-03822-s001.zip › plants-4002359-supplementary.pdf]

## Supplementary

## Climatic Drivers of Flowering Synchrony in 'Hass' Avocado under Tropical Andean Conditions

**Table S1.** Descriptive analysis of altitudinal climate structure and its phenological implications.

| Identification | Lot | Data | Lost | Mean | Standard error | Median | Mode | SD | Minimum | Maximum |
|---|---|---|---|---|---|---|---|---|---|---|
| Accumulated Rainfall (mm) | 3 | 386 | 1 | 5.4234 | 0.55912 | 0.400 | 0.0 | 10.9849 | 0.0 | 80.00 |
| | 5 | 1201 | 6 | 4.5087 | 0.24372 | 0.920 | 0.0 | 8.4463 | 0.0 | 80.00 |
| | 23 | 1061 | 16 | 3.4017 | 0.22213 | 0.490 | 0.0 | 7.2355 | 0.0 | 74.95 |
| Log(x+1) (rainfall) | 3 | 387 | 0 | 0.4263 | 0.02662 | 0.146 | 0.0 | 0.5238 | 0.0 | 1.908 |
| | 5 | 1207 | 0 | 0.4432 | 0.01345 | 0.276 | 0.0 | 0.4672 | 0.0 | 1.908 |
| | 23 | 1077 | 0 | 0.3606 | 0.01308 | 0.161 | 0.0 | 0.4292 | 0.0 | 1.881 |
| Average Ambient Humidity (%) | 3 | 387 | 0 | 81.0535 | 0.51683 | 84.310 | 80.880 | 10.1673 | 47.740 | 95.52 |
| | 5 | 1175 | 32 | 79.7167 | 0.29090 | 82.280 | 82.280 | 9.9716 | 37.140 | 96.61 |
| | 23 | 1003 | 74 | 87.3335 | 0.38357 | 90.360 | 100.0 | 12.1478 | 38.400 | 100.0 |
| (HR)* | 3 | 387 | 0 | 0.8105 | 0.00517 | 0.843 | 0.809 | 0.1017 | 0.477 | 0.955 |
| | 5 | 1175 | 32 | 0.7972 | 0.00291 | 0.823 | 0.823 | 0.0997 | 0.371 | 0.966 |
| | 23 | 1003 | 74 | 0.8733 | 0.00384 | 0.904 | 1.0 | 0.1215 | 0.384 | 1.0 |
| Average Ambient Temperature (°C) | 3 | 387 | 0 | 16.9538 | 0.08561 | 16.590 | 16.320 | 1.6841 | 13.510 | 25.150 |
| | 5 | 1178 | 29 | 15.2639 | 0.04976 | 15.070 | 14.100 | 1.7078 | 11.230 | 21.640 |
| | 23 | 1052 | 25 | 14.6891 | 0.05669 | 14.460 | 13.290 | 1.8386 | 10.160 | 25.150 |
| Maximum Ambient Temperature (°C) | 3 | 387 | 0 | 26.2898 | 0.20841 | 26.180 | 21.180 | 4.0999 | 15.700 | 36.780 |
| | 5 | 1178 | 29 | 21.9936 | 0.13748 | 21.390 | 25.450 | 4.7184 | 11.010 | 35.390 |
| | 23 | 1049 | 28 | 20.6617 | 0.14028 | 20.030 | 16.130 | 4.5434 | 10.820 | 35.140 |
| Minimum Ambient Temperature (°C) | 3 | 387 | 0 | 12.3088 | 0.05950 | 12.290 | 11.250 | 1.1704 | 8.890 | 16.530 |
| | 5 | 1178 | 29 | 11.8353 | 0.03411 | 11.695 | 11.910 | 1.1707 | 6.280 | 18.330 |
| | 23 | 1075 | 2 | 11.5451 | 0.04120 | 11.400 | 11.240 | 1.3507 | 7.880 | 23.410 |
| Average Wind Speed (m/s) | 3 | 349 | 38 | 0.4196 | 0.01610 | 0.410 | 0.0 | 0.3008 | 0.0 | 2.150 |
| | 5 | 1125 | 82 | 0.6451 | 0.01911 | 0.470 | 0.0 | 0.6410 | 0.0 | 4.310 |
| | 23 | 886 | 191 | 0.9570 | 0.03746 | 0.580 | 0.0 | 1.1151 | 0.0 | 8.220 |
| Maximum Wind Speed (m/s) | 3 | 349 | 38 | 2.6536 | 0.07110 | 2.800 | 0.0 | 1.3282 | 0.0 | 6.200 |
| | 5 | 1125 | 82 | 2.4497 | 0.06669 | 2.090 | 0.0 | 2.2369 | -7.000 | 16.600 |
| | 23 | 856 | 221 | 4.1185 | 0.10079 | 2.975 | 1.800 | 2.9488 | 0.200 | 18.200 |
| Minimum Wind Speed (m/s) | 3 | 349 | 38 | 0.0 | 0.0 | 0.0 | 0.0 | 0.0 | 0.0 | 0.0 |
| | 5 | 1125 | 82 | 0.0335 | 0.00544 | 0.0 | 0.0 | 0.1825 | 0.0 | 4.200 |
| | 23 | 1053 | 24 | 0.0626 | 0.00641 | 0.0 | 0.0 | 0.2081 | 0.0 | 2.800 |

* (HR): Humidity Relative was expressed as a ratio from 0 to 1.

**Table S2.** Correlation matrix of altitudinal climate structure and its phenological implications.

| Identification | Statistics | Accumulated Rainfall (mm) | Log(rainfall) | Average Ambient Humidity (%) | Relative humidity (%RH) | Average Ambient Temperature (°C) | Maximum Ambient Temperature (°C) | Minimum Ambient Temperature (°C) | Average Wind Speed (m/s) | Maximum Wind Speed (m/s) | Minimum Wind Speed (m/s) |
|---|---|---|---|---|---|---|---|---|---|---|---|
| Accumulated Rainfall (mm) | Correlation (r) | - | | | | | | | | | |
| | Valor *p* | - | | | | | | | | | |
| Log(x+1) (rainfall) | Correlation (r) | 0.837 | - | | | | | | | | |
| | Valor *p* | < 0.001 | - | | | | | | | | |
| Average Ambient Humidity (%) | Correlation (r) | 0.175 | 0.251 | - | | | | | | | |
| | Valor *p* | < 0.001 | < 0.001 | - | | | | | | | |
| (HR)* | Correlation (r) | 0.175 | 0.251 | 1.000 | - | | | | | | |
| | Valor *p* | < 0.001 | < 0.001 | < 0.001 | - | | | | | | |
| Average Ambient Temperature (°C) | Correlation (r) | -0.199 | -0.313 | -0.615 | -0.615 | - | | | | | |
| | Valor *p* | < 0.001 | < 0.001 | < 0.001 | < 0.001 | - | | | | | |
| Maximum Ambient Temperature (°C) | Correlation (r) | -0.122 | -0.206 | -0.449 | -0.449 | 0.824 | - | | | | |
| | Valor *p* | < 0.001 | < 0.001 | < 0.001 | < 0.001 | < 0.001 | - | | | | |
| Minimum Ambient Temperature (°C) | Correlation (r) | -0.037 | -0.112 | -0.321 | -0.321 | 0.508 | 0.106 | - | | | |
| | Valor *p* | 0.058 | < 0.001 | < 0.001 | < 0.001 | < 0.001 | < 0.001 | - | | | |
| Average Wind Speed (m/s) | Correlation (r) | -0.052 | -0.093 | -0.575 | -0.575 | 0.214 | 0.060 | 0.196 | - | | |
| | Valor *p* | 0.012 | < 0.001 | < 0.001 | < 0.001 | < 0.001 | 0.004 | < 0.001 | - | | |
| Maximum Wind Speed (m/s) | Correlation (r) | -0.064 | -0.133 | -0.449 | -0.449 | 0.242 | 0.131 | 0.154 | 0.843 | - | |
| | Valor *p* | 0.002 | < 0.001 | < 0.001 | < 0.001 | < 0.001 | < 0.001 | < 0.001 | < 0.001 | - | |
| Minimum Wind Speed (m/s) | Correlation (r) | -0.006 | 0.020 | -0.062 | -0.062 | -0.004 | -0.116 | 0.198 | 0.344 | 0.092 | - |
| | Valor *p* | 0.778 | 0.307 | 0.002 | 0.002 | 0.851 | < 0.001 | < 0.001 | < 0.001 | < 0.001 | - |

* (HR): Humidity Relative was expressed as a ratio from 0 to 1.

**Table S3.** Bartlett Sphericity Test of altitudinal climate structure and its phenological implications.

| $X^2$ | Df* | *P* Valor (%) |
|---|---|---|
| 4 | 45 | < 0.001 |

* Degrees of freedom (Df)

**Table S4.** Descriptive analysis of phenological dynamics of Hass avocado.

| Phenological stages | Cycle | Lot | Data | Lost | Mean | Median | SD | Minimum | Maximum |
|---|---|---|---|---|---|---|---|---|---|
| EF010 | 1 | 3 | 1073 | 7 | 57.21291 | 75.5 | 43.063 | 0.00 | 100.00 |
| | | 5 | 1199 | 1 | 79.92370 | 91.7 | 27.600 | 0.00 | 100.00 |
| | | 23 | 2031 | 3 | 45.71794 | 47.1 | 44.765 | 0.00 | 100.00 |
| | 2 | 3 | 840 | 0 | 67.34121 | 82.6 | 34.987 | 0.00 | 100.00 |
| | | 5 | 950 | 10 | 70.10231 | 88.0 | 37.890 | 0.00 | 100.00 |
| | | 23 | 0 | 0 | - | - | - | - | - |
| EF019 | 1 | 3 | 1073 | 7 | 0.33429 | 0.0 | 2.781 | 0.00 | 50.00 |
| | | 5 | 1198 | 2 | 0.05559 | 0.0 | 0.770 | 0.00 | 13.33 |
| | | 23 | 2031 | 3 | 0.34819 | 0.0 | 3.144 | 0.00 | 72.73 |
| | 2 | 3 | 839 | 1 | 0.31904 | 0.0 | 2.056 | 0.00 | 34.29 |
| | | 5 | 950 | 10 | 0.14921 | 0.0 | 2.628 | 0.00 | 71.43 |
| | | 23 | 0 | 0 | - | - | - | - | - |
| EF110 | 1 | 3 | 1073 | 7 | 255.929 | 0.0 | 11.522 | 0.00 | 100.00 |
| | | 5 | 1199 | 1 | 0.45395 | 0.0 | 2.733 | 0.00 | 50.00 |
| | | 23 | 2029 | 5 | 121.431 | 0.0 | 6.267 | 0.00 | 100.00 |
| | 2 | 3 | 839 | 1 | 0.65147 | 0.0 | 4.606 | 0.00 | 81.25 |
| | | 5 | 950 | 10 | 143.689 | 0.0 | 8.654 | 0.00 | 100.00 |
| | | 23 | 0 | 0 | - | - | - | - | - |
| EF115 | 1 | 3 | 1073 | 7 | 0.35178 | 0.0 | 3.251 | 0.00 | 62.50 |
| | | 5 | 1199 | 1 | 0.22904 | 0.0 | 2.359 | 0.00 | 62.50 |
| | | 23 | 2029 | 5 | 0.35881 | 0.0 | 3.372 | 0.00 | 100.00 |
| | 2 | 3 | 839 | 1 | 0.49347 | 0.0 | 4.063 | 0.00 | 75.00 |
| | | 5 | 950 | 10 | 170.895 | 0.0 | 9.901 | 0.00 | 100.00 |
| | | 23 | 0 | 0 | - | - | - | - | - |
| EF119 | 1 | 3 | 1073 | 7 | 0.20571 | 0.0 | 2.767 | 0.00 | 50.00 |
| | | 5 | 1199 | 1 | 0.02304 | 0.0 | 0.362 | 0.00 | 9.09 |
| | | 23 | 2029 | 5 | 0.02909 | 0.0 | 0.431 | 0.00 | 12.00 |
| | 2 | 3 | 839 | 1 | 0.10865 | 0.0 | 0.966 | 0.00 | 17.86 |
| | | 5 | 950 | 10 | 0.30251 | 0.0 | 3.056 | 0.00 | 75.00 |
| | | 23 | 0 | 0 | - | - | - | - | - |
| EF510 | 1 | 3 | 1073 | 7 | 0.05178 | 0.0 | 1.696 | 0.00 | 55.56 |
| | | 5 | 1199 | 1 | 0.01515 | 0.0 | 0.324 | 0.00 | 7.69 |
| | | 23 | 2029 | 5 | 0.05550 | 0.0 | 0.819 | 0.00 | 23.16 |
| | 2 | 3 | 839 | 1 | 0.00441 | 0.0 | 0.128 | 0.00 | 3.70 |
| | | 5 | 950 | 10 | 0.01754 | 0.0 | 0.541 | 0.00 | 16.67 |
| | | 23 | 0 | 0 | - | - | - | - | - |
| EF511 | 1 | 3 | 1073 | 7 | 0.10417 | 0.0 | 1.128 | 0.00 | 16.36 |
| | | 5 | 1199 | 1 | 0.20519 | 0.0 | 2.077 | 0.00 | 34.78 |
| | | 23 | 2029 | 5 | 330.316 | 0.0 | 9.807 | 0.00 | 66.67 |
| | 2 | 3 | 839 | 1 | 0.00000 | 0.0 | 0.000 | 0.00 | 0.00 |
| | | 5 | 950 | 10 | 0.00619 | 0.0 | 0.191 | 0.00 | 5.88 |
| | | 23 | 0 | 0 | - | - | - | - | - |
| EF512 | 1 | 3 | 1073 | 7 | 1.644.252 | 0.0 | 25.419 | 0.00 | 100.00 |
| | | 5 | 1199 | 1 | 996.066 | 0.0 | 16.548 | 0.00 | 100.00 |
| | | 23 | 2016 | 18 | 1.670.085 | 0.0 | 23.931 | 0.00 | 100.00 |
| | 2 | 3 | 839 | 1 | 541.356 | 0.0 | 12.146 | 0.00 | 100.00 |
| | | 5 | 950 | 10 | 845.486 | 0.0 | 17.485 | 0.00 | 100.00 |
| | | 23 | 0 | 0 | - | - | - | - | - |
| EF513 | 1 | 3 | 1073 | 7 | 860.889 | 0.0 | 17.654 | 0.00 | 100.00 |
| | | 5 | 1199 | 1 | 459.992 | 0.0 | 11.063 | 0.00 | 85.71 |
| | | 23 | 2017 | 17 | 1.494.800 | 0.0 | 21.970 | 0.00 | 100.00 |
| | 2 | 3 | 839 | 1 | 288.011 | 0.0 | 9.544 | 0.00 | 85.71 |
| | | 5 | 950 | 10 | 430.344 | 0.0 | 14.196 | 0.00 | 100.00 |
| | | 23 | 0 | 0 | - | - | - | - | - |
| EF514 | 1 | 3 | 1073 | 7 | 292.018 | 0.0 | 11.041 | 0.00 | 100.00 |
| | | 5 | 1199 | 1 | 114.583 | 0.0 | 5.228 | 0.00 | 71.43 |
| | | 23 | 2027 | 7 | 415.805 | 0.0 | 10.785 | 0.00 | 100.00 |
| | 2 | 3 | 839 | 1 | 123.927 | 0.0 | 5.136 | 0.00 | 73.83 |
| | | 5 | 950 | 10 | 110.117 | 0.0 | 5.610 | 0.00 | 75.00 |
| | | 23 | 0 | 0 | - | - | - | - | - |
| EF515 | 1 | 3 | 1073 | 7 | 173.079 | 0.0 | 7.750 | 0.00 | 100.00 |

| | | | | | | | | | |
|---|---|---|---|---|---|---|---|---|---|
| | | 5 | 1199 | 1 | 0.72599 | 0.0 | 3.856 | 0.00 | 51.72 |
| | | 23 | 2027 | 7 | 251.377 | 0.0 | 8.614 | 0.00 | 88.24 |
| | 2 | 3 | 839 | 1 | 209.285 | 0.0 | 7.013 | 0.00 | 100.00 |
| | | 5 | 950 | 10 | 115.773 | 0.0 | 5.454 | 0.00 | 66.67 |
| | | 23 | 0 | 0 | - | - | - | - | - |
| EF517 | 1 | 3 | 1073 | 7 | 0.87597 | 0.0 | 5.718 | 0.00 | 100.00 |
| | | 5 | 1199 | 1 | 0.46955 | 0.0 | 3.893 | 0.00 | 65.00 |
| | | 23 | 2028 | 6 | 161.478 | 0.0 | 7.436 | 0.00 | 91.67 |
| | 2 | 3 | 839 | 1 | 156.973 | 0.0 | 6.381 | 0.00 | 59.09 |
| | | 5 | 950 | 10 | 116.647 | 0.0 | 6.238 | 0.00 | 81.82 |
| | | 23 | 0 | 0 | - | - | - | - | - |
| EF518 | 1 | 3 | 1073 | 7 | 311.047 | 0.0 | 12.037 | 0.00 | 100.00 |
| | | 5 | 1199 | 1 | 0.54798 | 0.0 | 3.379 | 0.00 | 63.64 |
| | | 23 | 2026 | 8 | 420.905 | 0.0 | 13.586 | 0.00 | 100.00 |
| | 2 | 3 | 839 | 1 | 216.595 | 0.0 | 8.647 | 0.00 | 92.63 |
| | | 5 | 950 | 10 | 103.309 | 0.0 | 5.431 | 0.00 | 57.14 |
| | | 23 | 0 | 0 | - | - | - | - | - |
| EF610 | 1 | 3 | 1073 | 7 | 199.824 | 0.0 | 10.711 | 0.00 | 100.00 |
| | | 5 | 1199 | 1 | 0.57932 | 0.0 | 4.599 | 0.00 | 63.64 |
| | | 23 | 2026 | 8 | 135.149 | 0.0 | 8.009 | 0.00 | 100.00 |
| | 2 | 3 | 839 | 1 | 403.813 | 0.0 | 13.287 | 0.00 | 100.00 |
| | | 5 | 950 | 10 | 280.940 | 0.0 | 12.619 | 0.00 | 100.00 |
| | | 23 | 0 | 0 | - | - | - | - | - |
| EF617 | 1 | 3 | 1073 | 7 | 273.338 | 0.0 | 13.348 | 0.00 | 100.00 |
| | | 5 | 1199 | 1 | 0.03452 | 0.0 | 0.442 | 0.00 | 8.11 |
| | | 23 | 2024 | 10 | 183.799 | 0.0 | 9.557 | 0.00 | 100.00 |
| | 2 | 3 | 839 | 1 | 959.753 | 0.0 | 24.337 | 0.00 | 100.00 |
| | | 5 | 950 | 10 | 403.349 | 0.0 | 16.086 | 0.00 | 100.00 |
| | | 23 | 0 | 0 | - | - | - | - | - |
| EF711 | 1 | 3 | 1073 | 7 | 0.25822 | 0.0 | 2.895 | 0.00 | 50.00 |
| | | 5 | 1199 | 1 | 0.26499 | 0.0 | 1.526 | 0.00 | 20.45 |
| | | 23 | 2029 | 5 | 0.19602 | 0.0 | 1.497 | 0.00 | 28.57 |
| | 2 | 3 | 839 | 1 | 0.83654 | 0.0 | 5.283 | 0.00 | 87.76 |
| | | 5 | 950 | 10 | 0.17471 | 0.0 | 2.102 | 0.00 | 46.67 |
| | | 23 | 0 | 0 | - | - | - | - | - |
| EF712 | 1 | 3 | 1073 | 7 | 0.05865 | 0.0 | 0.772 | 0.00 | 14.29 |
| | | 5 | 1199 | 1 | 0.33803 | 0.0 | 1.635 | 0.00 | 20.00 |
| | | 23 | 2029 | 5 | 0.18610 | 0.0 | 1.227 | 0.00 | 14.29 |
| | 2 | 3 | 839 | 1 | 0.39485 | 0.0 | 2.689 | 0.00 | 48.57 |
| | | 5 | 950 | 10 | 0.29080 | 0.0 | 2.005 | 0.00 | 37.21 |
| | | 23 | 0 | 0 | - | - | - | - | - |
| EF715 | 1 | 3 | 1073 | 7 | 0.19405 | 0.0 | 1.949 | 0.00 | 50.00 |
| | | 5 | 1199 | 1 | 0.31378 | 0.0 | 2.007 | 0.00 | 25.00 |
| | | 23 | 2029 | 5 | 0.19282 | 0.0 | 1.527 | 0.00 | 36.36 |
| | 2 | 3 | 839 | 1 | 0.89215 | 0.0 | 3.368 | 0.00 | 42.86 |
| | | 5 | 950 | 10 | 175.123 | 0.0 | 6.219 | 0.00 | 100.00 |
| | | 23 | 0 | 0 | - | - | - | - | - |
| EF719 | 1 | 3 | 1073 | 7 | 0.20110 | 0.0 | 1.732 | 0.00 | 33.33 |
| | | 5 | 1199 | 1 | 0.11383 | 0.0 | 1.083 | 0.00 | 25.00 |
| | | 23 | 2029 | 5 | 0.25549 | 0.0 | 2.318 | 0.00 | 66.67 |
| | 2 | 3 | 839 | 1 | 0.00000 | 0.0 | 0.000 | 0.00 | 0.00 |
| | | 5 | 950 | 10 | 0.00000 | 0.0 | 0.000 | 0.00 | 0.00 |
| | | 23 | 0 | 0 | - | - | - | - | - |

**Table S5.** Partial correlation of phenological dynamics of Hass avocado.

| Identification | Cycle | Statistics | EF010 | EF019 | EF110 | EF115 | EF119 | EF510 | EF511 | EF512 | EF513 | EF514 | EF515 | EF517 | EF518 | EF610 | EF617 | EF711 | EF712 | EF715 | EF719 |
|---|---|---|---|---|---|---|---|---|---|---|---|---|---|---|---|---|---|---|---|---|---|
| EF010 | 1 | Correlation (r) | - | | | | | | | | | | | | | | | | | | |
| | | Valor *p* | - | | | | | | | | | | | | | | | | | | |
| | 2 | Correlation (r) | - | | | | | | | | | | | | | | | | | | |
| | | Valor *p* | - | | | | | | | | | | | | | | | | | | |
| EF019 | 1 | Correlation (r) | -0.038 | - | | | | | | | | | | | | | | | | | |
| | | Valor *p* | 0.003 | - | | | | | | | | | | | | | | | | | |
| | 2 | Correlation (r) | -0.037 | - | | | | | | | | | | | | | | | | | |
| | | Valor *p* | 0.004 | - | | | | | | | | | | | | | | | | | |
| EF110 | 1 | Correlation (r) | -0.212 | 0.053 | - | | | | | | | | | | | | | | | | |
| | | Valor *p* | < .001 | < .001 | - | | | | | | | | | | | | | | | | |
| | 2 | Correlation (r) | -0.219 | 0.037 | - | | | | | | | | | | | | | | | | |
| | | Valor *p* | < .001 | 0.004 | - | | | | | | | | | | | | | | | | |
| EF115 | 1 | Correlation (r) | -0.120 | 0.053 | 0.072 | - | | | | | | | | | | | | | | | |
| | | Valor *p* | < .001 | < .001 | < .001 | - | | | | | | | | | | | | | | | |
| | 2 | Correlation (r) | -0.131 | 0.037 | 0.179 | - | | | | | | | | | | | | | | | |
| | | Valor *p* | < .001 | 0.004 | < .001 | - | | | | | | | | | | | | | | | |
| EF119 | 1 | Correlation (r) | -0.052 | -0.004 | 0.016 | 0.036 | - | | | | | | | | | | | | | | |
| | | Valor *p* | < .001 | 0.762 | 0.209 | 0.005 | - | | | | | | | | | | | | | | |
| | 2 | Correlation (r) | -0.050 | 0.003 | 0.029 | 0.188 | - | | | | | | | | | | | | | | |
| | | Valor *p* | < .001 | 0.817 | 0.026 | < .001 | - | | | | | | | | | | | | | | |
| EF510 | 1 | Correlation (r) | -0.030 | -0.000 | 0.026 | 0.001 | 0.000 | - | | | | | | | | | | | | | |
| | | Valor *p* | 0.018 | 0.984 | 0.041 | 0.919 | 0.993 | - | | | | | | | | | | | | | |
| | 2 | Correlation (r) | -0.039 | 0.010 | 0.009 | 0.020 | 0.022 | - | | | | | | | | | | | | | |
| | | Valor *p* | 0.003 | 0.447 | 0.485 | 0.115 | 0.088 | - | | | | | | | | | | | | | |
| EF511 | 1 | Correlation (r) | -0.122 | -0.006 | -0.026 | -0.020 | -0.008 | 0.012 | - | | | | | | | | | | | | |
| | | Valor *p* | < .001 | 0.633 | 0.039 | 0.119 | 0.558 | 0.369 | - | | | | | | | | | | | | |
| | 2 | Correlation (r) | -0.111 | 0.041 | -0.056 | -0.026 | 0.004 | 0.028 | - | | | | | | | | | | | | |
| | | Valor *p* | < .001 | 0.001 | < .001 | 0.043 | 0.773 | 0.032 | - | | | | | | | | | | | | |
| EF512 | 1 | Correlation (r) | -0.521 | -0.026 | -0.040 | -0.018 | -0.024 | 0.010 | 0.010 | - | | | | | | | | | | | |
| | | Valor *p* | < .001 | 0.043 | 0.002 | 0.150 | 0.060 | 0.414 | 0.414 | - | | | | | | | | | | | |
| | 2 | Correlation (r) | -0.519 | -0.010 | -0.048 | 0.001 | -0.029 | 0.024 | 0.024 | - | | | | | | | | | | | |
| | | Valor *p* | < .001 | 0.421 | < .001 | 0.923 | 0.025 | 0.065 | 0.065 | - | | | | | | | | | | | |
| EF513 | 1 | Correlation (r) | -0.579 | 0.041 | 0.058 | 0.009 | -0.005 | 0.005 | 0.005 | 0.173 | - | | | | | | | | | | |
| | | Valor *p* | < .001 | 0.001 | < .001 | 0.483 | 0.678 | 0.688 | 0.688 | < .001 | - | | | | | | | | | | |
| | 2 | Correlation (r) | -0.591 | -0.015 | 0.088 | 0.020 | -0.020 | 0.019 | 0.019 | 0.294 | - | | | | | | | | | | |
| | | Valor *p* | < .001 | 0.246 | < .001 | 0.118 | 0.118 | 0.143 | 0.143 | < .001 | - | | | | | | | | | | |
| EF514 | 1 | Correlation (r) | -0.349 | -0.020 | 0.027 | -0.008 | -0.017 | -0.003 | -0.003 | 0.020 | 0.211 | - | | | | | | | | | |
| | | Valor *p* | < .001 | 0.128 | 0.035 | 0.546 | 0.197 | 0.827 | 0.827 | 0.123 | < .001 | - | | | | | | | | | |
| | 2 | Correlation (r) | -0.367 | -0.034 | 0.049 | 0.000 | -0.022 | 0.027 | 0.027 | 0.101 | 0.363 | - | | | | | | | | | |
| | | Valor *p* | < .001 | 0.008 | < .001 | 0.996 | 0.082 | 0.038 | 0.038 | < .001 | < .001 | - | | | | | | | | | |
| EF515 | 1 | Correlation (r) | -0.305 | -0.008 | 0.030 | 0.006 | 0.008 | -0.001 | -0.001 | 0.040 | 0.146 | 0.148 | - | | | | | | | | |
| | | Valor *p* | < .001 | 0.513 | 0.019 | 0.624 | 0.523 | 0.957 | 0.957 | 0.002 | < .001 | < .001 | - | | | | | | | | |
| | 2 | Correlation (r) | -0.317 | -0.029 | 0.048 | 0.046 | 0.012 | 0.061 | 0.061 | 0.081 | 0.245 | 0.257 | - | | | | | | | | |
| | | Valor *p* | < .001 | 0.025 | < .001 | < .001 | 0.361 | < .001 | < .001 | < .001 | < .001 | < .001 | - | | | | | | | | |
| EF517 | 1 | Correlation (r) | -0.237 | 0.005 | 0.024 | 0.057 | -0.001 | -0.007 | -0.007 | 0.010 | 0.093 | 0.072 | 0.134 | - | | | | | | | |
| | | Valor *p* | < .001 | 0.670 | 0.063 | < .001 | 0.921 | 0.588 | 0.588 | 0.452 | < .001 | < .001 | < .001 | - | | | | | | | |
| | 2 | Correlation (r) | -0.248 | -0.020 | 0.057 | 0.057 | -0.002 | -0.003 | -0.003 | 0.044 | 0.152 | 0.157 | 0.240 | - | | | | | | | |
| | | Valor *p* | < .001 | 0.114 | < .001 | < .001 | 0.855 | 0.787 | 0.787 | < .001 | < .001 | < .001 | < .001 | - | | | | | | | |
| EF518 | 1 | Correlation (r) | -0.329 | -0.022 | 0.109 | 0.006 | -0.008 | 0.002 | 0.002 | -0.038 | 0.112 | 0.125 | 0.112 | 0.078 | - | | | | | | |
| | | Valor *p* | < .001 | 0.088 | < .001 | 0.656 | 0.544 | 0.859 | 0.859 | 0.003 | < .001 | < .001 | < .001 | < .001 | - | | | | | | |
| | 2 | Correlation (r) | -0.327 | -0.041 | 0.145 | 0.017 | 0.026 | 0.022 | 0.022 | -0.025 | 0.201 | 0.177 | 0.240 | 0.182 | - | | | | | | |
| | | Valor *p* | < .001 | 0.001 | < .001 | 0.192 | 0.044 | 0.085 | 0.085 | 0.050 | < .001 | < .001 | < .001 | < .001 | - | | | | | | |

| Identification | Cycle | Statistics | EF010 | EF019 | EF110 | EF115 | EF119 | EF510 | EF511 | EF512 | EF513 | EF514 | EF515 | EF517 | EF518 | EF610 | EF617 | EF711 | EF712 | EF715 | EF719 |
|---|---|---|---|---|---|---|---|---|---|---|---|---|---|---|---|---|---|---|---|---|---|
| EF610 | 1 | Correlation (r) | -0.249 | -0.015 | 0.006 | 0.018 | 0.003 | -0.006 | -0.006 | -0.044 | -0.014 | -0.006 | 0.059 | 0.047 | 0.047 | - | | | | | |
| | | Valor *p* | < .001 | 0.237 | 0.647 | 0.152 | 0.829 | 0.623 | 0.623 | < .001 | 0.268 | 0.614 | < .001 | < .001 | < .001 | - | | | | | |
| | 2 | Correlation (r) | -0.245 | -0.028 | 0.036 | 0.036 | 0.035 | -0.004 | -0.004 | -0.059 | 0.017 | 0.019 | 0.124 | 0.096 | 0.096 | - | | | | | |
| | | Valor *p* | < .001 | 0.027 | 0.006 | 0.005 | 0.006 | 0.726 | 0.726 | < .001 | 0.190 | 0.143 | < .001 | < .001 | < .001 | - | | | | | |
| EF617 | 1 | Correlation (r) | -0.273 | 0.009 | 0.000 | 0.020 | 0.058 | -0.004 | -0.004 | -0.092 | -0.024 | -0.037 | -0.013 | -0.025 | -0.025 | 0.066 | - | | | | |
| | | Valor *p* | < .001 | 0.474 | 0.988 | 0.124 | < .001 | 0.739 | 0.739 | < .001 | 0.064 | 0.004 | 0.311 | 0.047 | 0.047 | < .001 | - | | | | |
| | 2 | Correlation (r) | -0.250 | -0.040 | 0.017 | 0.041 | 0.097 | -0.006 | -0.006 | -0.122 | -0.016 | -0.049 | 0.014 | -0.012 | -0.012 | 0.215 | - | | | | |
| | | Valor *p* | < .001 | 0.002 | 0.195 | 0.001 | < .001 | 0.656 | 0.656 | < .001 | 0.200 | < .001 | 0.262 | 0.332 | 0.332 | < .001 | - | | | | |
| EF711 | 1 | Correlation (r) | -0.029 | -0.008 | 0.015 | -0.013 | 0.008 | 0.001 | 0.001 | -0.061 | -0.031 | -0.024 | -0.027 | -0.017 | -0.017 | -0.013 | -0.013 | - | | | |
| | | Valor *p* | 0.022 | 0.525 | 0.240 | 0.318 | 0.527 | 0.933 | 0.933 | < .001 | 0.015 | 0.065 | 0.037 | 0.180 | 0.180 | 0.298 | 0.298 | - | | | |
| | 2 | Correlation (r) | 0.016 | 0.020 | -0.022 | -0.032 | 0.022 | 0.052 | 0.052 | -0.104 | -0.104 | -0.057 | -0.048 | -0.043 | -0.043 | -0.036 | -0.036 | - | | | |
| | | Valor *p* | 0.211 | 0.118 | 0.086 | 0.011 | 0.081 | < .001 | < .001 | < .001 | < .001 | < .001 | < .001 | < .001 | < .001 | 0.005 | 0.005 | - | | | |
| EF712 | 1 | Correlation (r) | 0.048 | -0.011 | -0.018 | -0.012 | -0.009 | -0.005 | -0.005 | -0.065 | -0.052 | -0.024 | -0.008 | -0.018 | -0.018 | -0.006 | -0.006 | 0.094 | - | | |
| | | Valor *p* | < .001 | 0.391 | 0.154 | 0.361 | 0.469 | 0.671 | 0.671 | < .001 | < .001 | 0.056 | 0.530 | 0.157 | 0.157 | 0.646 | 0.646 | < .001 | - | | |
| | 2 | Correlation (r) | 0.038 | 0.006 | -0.025 | -0.020 | -0.021 | -0.011 | -0.011 | -0.078 | -0.090 | -0.051 | -0.041 | -0.034 | -0.034 | -0.028 | -0.028 | 0.166 | - | | |
| | | Valor *p* | 0.003 | 0.625 | 0.053 | 0.116 | 0.102 | 0.389 | 0.389 | < .001 | < .001 | < .001 | 0.001 | 0.008 | 0.008 | 0.031 | 0.031 | < .001 | - | | |
| EF715 | 1 | Correlation (r) | -0.104 | -0.002 | 0.006 | 0.008 | 0.041 | -0.006 | -0.006 | -0.047 | -0.027 | -0.004 | 0.032 | 0.056 | 0.056 | 0.074 | 0.074 | 0.014 | -0.010 | - | |
| | | Valor *p* | < .001 | 0.872 | 0.650 | 0.511 | 0.001 | 0.649 | 0.649 | < .001 | 0.032 | 0.766 | 0.011 | < .001 | < .001 | < .001 | < .001 | 0.290 | 0.441 | - | |
| | 2 | Correlation (r) | -0.082 | 0.000 | -0.013 | 0.010 | 0.022 | -0.004 | -0.004 | -0.069 | -0.033 | 0.017 | 0.070 | 0.078 | 0.078 | 0.096 | 0.096 | -0.008 | -0.023 | - | |
| | | Valor *p* | < .001 | 0.993 | 0.321 | 0.421 | 0.092 | 0.781 | 0.781 | < .001 | 0.011 | 0.185 | < .001 | < .001 | < .001 | < .001 | < .001 | 0.522 | 0.076 | - | |
| EF719 | 1 | Correlation (r) | -0.044 | 0.017 | 0.016 | -0.009 | -0.006 | -0.003 | -0.003 | -0.020 | 0.004 | 0.042 | -0.012 | -0.008 | -0.008 | -0.009 | -0.009 | -0.005 | 0.018 | 0.041 | - |
| | | Valor *p* | < .001 | 0.195 | 0.217 | 0.464 | 0.652 | 0.791 | 0.791 | 0.116 | 0.727 | 0.001 | 0.330 | 0.508 | 0.508 | 0.489 | 0.489 | 0.714 | 0.158 | 0.001 | - |
| | 2 | Correlation (r) | -0.006 | 0.065 | 0.017 | -0.022 | -0.015 | -0.008 | -0.008 | -0.069 | -0.037 | -0.011 | -0.021 | -0.019 | -0.019 | -0.014 | -0.014 | 0.047 | 0.069 | 0.048 | - |
| | | Valor *p* | 0.623 | < .001 | 0.195 | 0.086 | 0.228 | 0.526 | 0.526 | < .001 | 0.004 | 0.395 | 0.094 | 0.131 | 0.131 | 0.272 | 0.272 | < .001 | < .001 | < .001 | - |

**Table S6.** Welch's ANOVA of phenological dynamics of Hass avocado.

| Components | F | Df[1] | DF[2] | *p* |
|---|---|---|---|---|
| Component 3, anthesis | 200.9 | 2 | 3688 | < 0.001 |
| Component 2, floral development | 44.0 | 2 | 3846 | < 0.001 |
| Component 1, floral morphogenesis | 159.2 | 2 | 3676 | < 0.001 |
| EF010 | 305.4 | 2 | 3923 | < 0.001 |

**Table S7.** Welch's ANOVA and Tukey post hoc tests of phenological dynamics of Hass avocado.

| Components | DM* | *p* |
|---|---|---|
| Component 3, anthesis | 0.285 | < 0.001 |
| Component 2, floral development | -0.402 | < 0.001 |
| Component 1, floral morphogenesis | 0.435 | < 0.001 |
| EF010 | -10.7 | < 0.001 |

* Difference of means (DM)

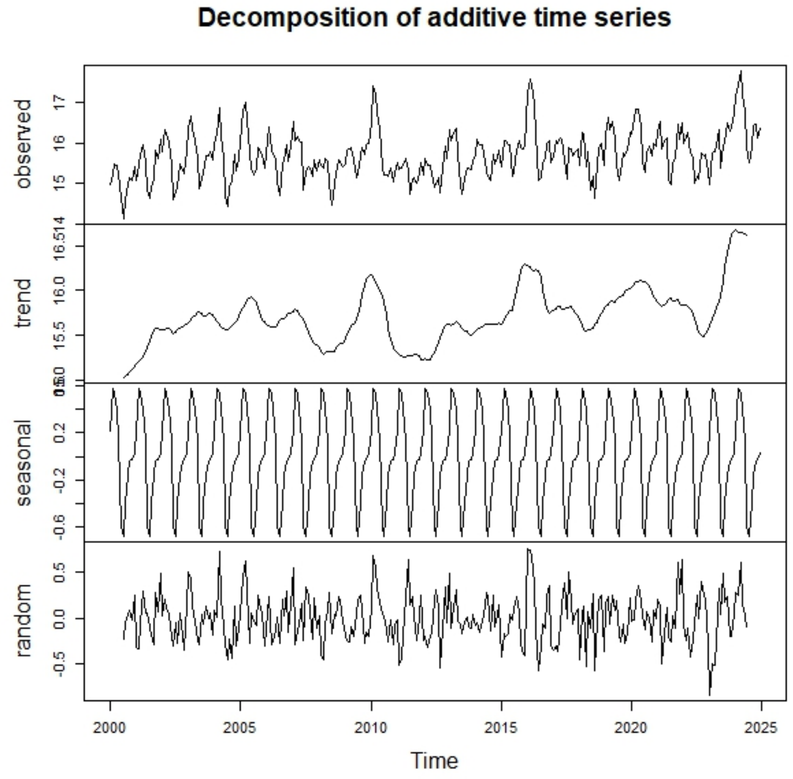


**Figure S1.** Seasonal–Trend decomposition using Loess (STL).

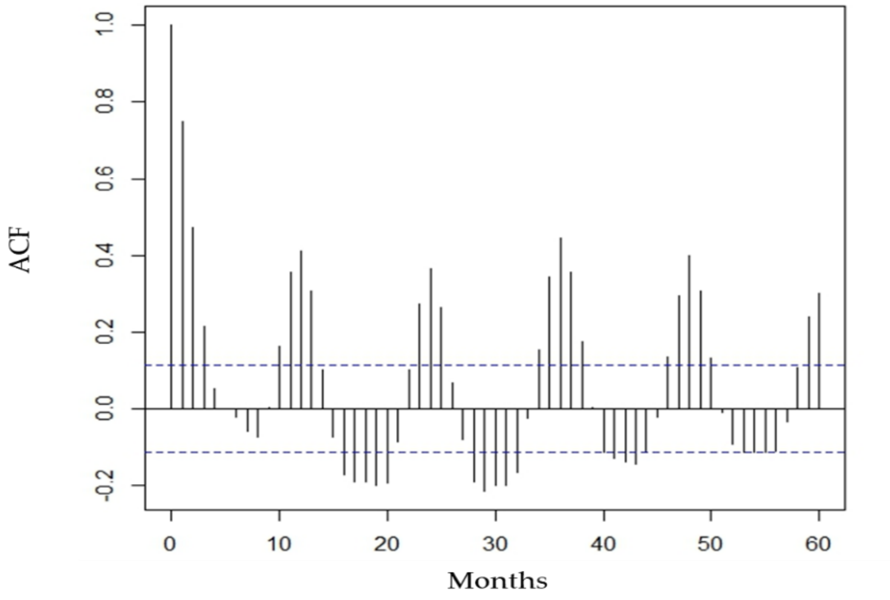


**Figure S2.** Autocorrelation function (ACF).

**Table S8.** Circular analysis applied today of year (DOY).

| Plots | Stages | Average | (ϱ) | Average Grades | Average DOY |
|---|---|---|---|---|---|
| L3 | Anthesis | 58.76 | 0.52 | 58.76 | 59,58 |
| | Floral development | 149.0 | 0.24 | 149.0 | 151,07 |
| | EF010 | 93.22 | 0.24 | 93.22 | 94,52 |
| | Floral morphogenesis | 177.55 | 0.36 | 177.55 | 180,02 |
| L5 | Anthesis | 81.75 | 0.64 | 81.75 | 82,89 |
| | Floral development | 157.56 | 0.34 | 157.56 | 159,75 |
| | EF010 | 125.90 | 0.38 | 125.90 | 127,65 |
| | Floral morphogenesis | 144.67 | 0.37 | 144.67 | 146,67 |
| L23 | Anthesis | -167.80 | 0.94 | -167.80 | -170,13 |
| | Floral development | 163.60 | 0.86 | 163.60 | 165,87 |
| | EF010 | 113.75 | 0.90 | 113.75 | 115,33 |
| | Floral morphogenesis | 141.39 | 0.83 | 141.39 | 143,35 |

**Table S9.** Description of phenological stages.

| Phenological stages | Description |
|---|---|
| EF010 | Vegetative buds dormant |
| EF019 | End of bud break |
| EF110 | First leaves separating |
| EF115 | Expanded leaves |
| EF119 | All leaves unfolded and fully expanded |
| EF510 | Reproductive buds dormant |
| EF511 | Beginning of reproductive bud swell |
| EF512 | End of reproductive bud swell |
| EF513 | Reproductive bud break |
| EF514 | Compound and separate inflorescence |

| | |
|---|---|
| EF515 | Inflorescences 50% of final length |
| EF517 | Inflorescences 70% of final length |
| EF518 | Inflorescence |
| EF610 | First flowers open |
| EF617 | open Flowers |
| EF711 | Initial ovary growth |
| EF712 | First fruitlet abscission |
| EF715 | 50% of final fruit size |
| EF719 | 90% or more of final fruit size |

Adapted from Phenological growth stages of avocado (Persea americana) according to the BBCH scale (Alcaraz et al., 2013).

**Table S10.** Welch's ANOVA of Altitudinal climate structure.

| **Components** | **F** | **Gl[1]** | **Gl[2]** | ***p*** |
|---|---|---|---|---|
| Factor 1 Temperature | 251.4 | 2 | 1114 | < 0.001 |
| Factor 2 Wind | 117.6 | 2 | 1224 | < 0.001 |
| Factor 3 Water | 37.7 | 2 | 1001 | < 0.001 |

**Table S11.** The Tukey post hoc test for temperature among months.

| Identification | Statistics | Jan | Feb | Mar | Apr | May | Jun | Jul | Aug | Sep | Oct | Nov | Dec |
|---|---|---|---|---|---|---|---|---|---|---|---|---|---|
| Jan | Valor *p* | - | <.001 | <.001 | <.001 | 0.993 | <.001 | <.001 | <.001 | <.001 | <.001 | <.001 | 0.075 |
| Feb | Valor *p* | <.001 | - | 1.000 | 0.016 | <.001 | <.001 | <.001 | <.001 | <.001 | <.001 | <.001 | <.001 |
| Mar | Valor *p* | <.001 | 1.000 | - | 0.050 | <.001 | <.001 | <.001 | <.001 | <.001 | <.001 | <.001 | <.001 |
| Apr | Valor *p* | <.001 | 0.016 | 0.050 | - | <.001 | <.001 | <.001 | <.001 | <.001 | <.001 | <.001 | <.001 |
| May | Valor *p* | 0.993 | <.001 | <.001 | <.001 | - | <.001 | <.001 | <.001 | <.001 | 0.006 | 0.012 | 0.698 |
| Jun | Valor *p* | <.001 | <.001 | <.001 | <.001 | <.001 | - | 0.327 | <.001 | <.001 | <.001 | <.001 | <.001 |
| Jul | Valor *p* | <.001 | <.001 | <.001 | <.001 | <.001 | 0.327 | - | <.001 | <.001 | <.001 | <.001 | <.001 |
| Aug | Valor *p* | <.001 | <.001 | <.001 | <.001 | <.001 | <.001 | <.001 | - | <.001 | <.001 | <.001 | <.001 |
| Sep | Valor *p* | <.001 | <.001 | <.001 | <.001 | <.001 | <.001 | <.001 | <.001 | - | 0.165 | 0.118 | <.001 |
| Oct | Valor *p* | <.001 | <.001 | <.001 | <.001 | 0.006 | <.001 | <.001 | <.001 | 0.165 | - | 1.000 | 0.766 |
| Nov | Valor *p* | <.001 | <.001 | <.001 | <.001 | 0.012 | <.001 | <.001 | <.001 | 0.118 | 1.000 | - | 0.861 |
| Dec | Valor *p* | 0.075 | <.001 | <.001 | <.001 | 0.698 | <.001 | <.001 | <.001 | <.001 | 0.766 | 0.861 | - |
